# CAVIN3 deficiency promotes vascular normalization in ocular neovascular disease via ERK/JAG1 signaling pathway

**DOI:** 10.1172/jci.insight.187836

**Published:** 2025-05-08

**Authors:** Weiqi Li, Yeran Zhang, Hongjing Zhu, Na Su, Ruxu Sun, Xiying Mao, Qin Yang, Songtao Yuan

**Affiliations:** 1 Department of Ophthalmology, The First Affiliated Hospital of Nanjing Medical University, Nanjing, China.; 2 Department of Ophthalmology, Children’s Hospital of Nanjing Medical University, Nanjing, China.

**Keywords:** Angiogenesis, Ophthalmology, Endothelial cells, Retinopathy

## Abstract

Multiple members of the caveolae-associated protein (Cavin) family are implicated in angiogenesis. However, the specific role of CAVIN3 in pathological angiogenesis within the eye remains unclear. The present study demonstrated that CAVIN3 knockdown in endothelial cells (ECs) promoted vascular normalization in ocular pathological neovascularization. Elevated CAVIN3 expression was observed in the ECs of retinal pigment epithelium/choroid complexes from patients with neovascular age-related macular degeneration and fibrovascular membranes from patients with proliferative diabetic retinopathy. Additionally, upregulated Cavin3 expression was detected in laser-induced choroidal neovascularization (CNV) and oxygen-induced retinopathy (OIR) mouse models. In both OIR and CNV mice, Cavin3 knockdown inhibited pathological neovascularization. Cavin3 deficiency further disrupted EC proliferation and vascular sprouting, thereby promoting vascular normalization by partially restoring microenvironmental hypoxia and reestablishing pericyte-EC interactions. Mechanistically, we demonstrated that zinc finger E-box–binding homeobox 1 (ZEB1) regulated *CAVIN3* transcription in ECs under hypoxic conditions. CAVIN3 deficiency modulated pathological vascularization by inhibiting ERK phosphorylation, which downregulated jagged 1 (JAG1) expression. Conclusively, this study elucidated the protective role of endothelial CAVIN3 deficiency in pathological neovascularization models, addressing a gap in understanding the regulatory role of Cavins in angiogenesis. These findings suggested a therapeutic direction for ocular neovascular diseases.

## Abstract 2

We found that endothelial CAVIN3 deficiency promotes vascular normalization in ocular pathological neovascularization. This effect is mediated through the inhibition of ERK phosphorylation and JAG1.

## Graphical abstract

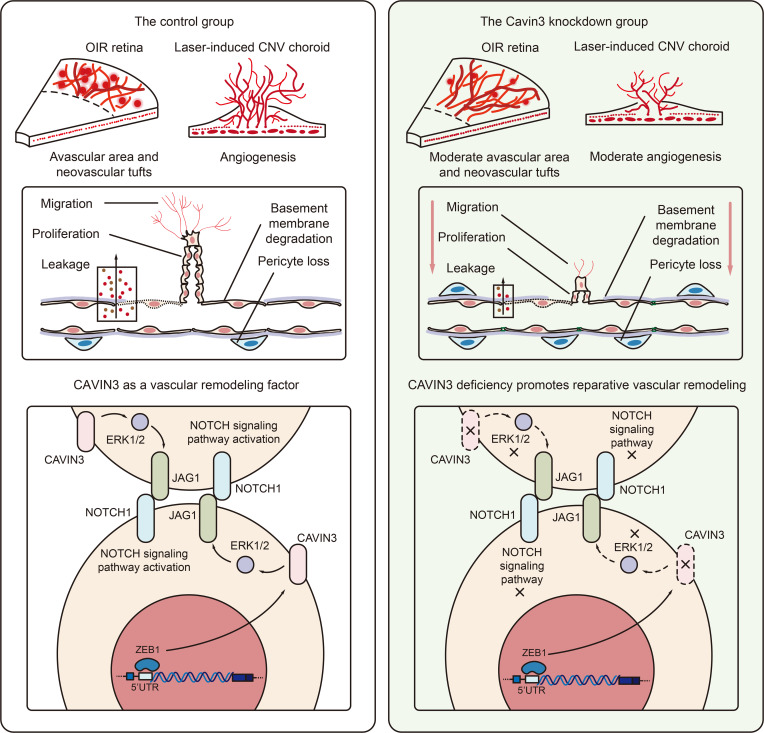

## Introduction

Pathological neovascularization is a prevalent characteristic of several ocular diseases, including diabetic retinopathy (DR) (1), retinopathy of prematurity (ROP) (2), and neovascular age-related macular degeneration (nAMD) (3). Vascular normalization refers to the restoration of blood vessels and their surrounding microenvironment from a pathological to a normal state (4, 5). Antiangiogenic therapy enhances vascular normalization by restoring equilibrium between pro- and antiangiogenic signaling, leading to a vasculature with improved architecture and function (6, 7). A marked increase in VEGF expression contributes to pathological neovascularization (8). Although intravitreal injections of anti-VEGF drugs are central to the clinical management of these neovascular eye diseases, many patients experience recurrence of neovascular lesions or exhibit resistance to treatment (9, 10). Thus, fresh insights into the pathology of pathological neovascularization are needed to identify novel therapeutic targets for more effective treatment strategies.

Endothelial cells (ECs) are key targets and are mostly studied in these pathological processes (11, 12). Increased EC sprouting and proliferation are the primary cellular events driving pathological angiogenesis (13). Disruption of tight junctions between ECs can lead to increased vascular permeability (12, 14). EC dysfunction prevents interaction with other vascular constituent cells, such as pericytes, further contributing to pathological vascular leakage (15). Additionally, the enhanced conversion of ECs into tip cells and the degradation of the vascular basement membrane can facilitate vascular sprouting (16). Therefore, it is essential to investigate how ECs participate in the angiogenic pathway and identify potential therapeutic targets.

Caveolae-associated protein 3 (CAVIN3) is a member of the Cavin family and plays a crucial role in regulating the formation and stabilization of caveolae in the cytoplasmic membrane (17). A subset of Cavin family members are associated with angiogenesis, but the role of CAVIN3 remains unclear (18–20). When subjected to mechanical stress, these CAVIN3-positive caveolae can disassemble, releasing CAVIN3 into the cytoplasm to propagate signaling pathways (21). We speculate that CAVIN3 may be involved in vascular outgrowth.

In this study, the role of CAVIN3 in the regulation of pathological neovascularization has been identified. We evaluated upregulated CAVIN3 expression in patients with proliferative DR (PDR) and nAMD. Additionally, the upregulation of Cavin3 in ECs was observed in oxygen-induced retinopathy (OIR) and choroidal neovascularization (CNV) mouse models. Furthermore, CAVIN3 deficiency promoted the regression of pathological vessels and vascular normalization by inhibiting EC proliferation and tip cell formation. This deficiency locally restored microenvironmental hypoxia and reestablished blood-retinal barrier (BRB) integrity, critical factors contributing to pathological vessel normalization. Under hypoxic conditions, we also found the knockdown of CAVIN3 restored hypoxia-induced proliferation, migration, and tube formation in ECs. Mechanistically, CAVIN3 knockdown inhibited ERK phosphorylation and further downregulated jagged 1 (JAG1) expression to regulate angiogenesis. Moreover, zinc finger E-box–binding homeobox 1 (ZEB1) positively regulated CAVIN3 expression under hypoxia. Therefore, targeting CAVIN3 may represent a therapeutic approach for the treatment of neovascular ocular diseases.

## Results

### CAVIN3 is upregulated in clinical ocular neovascular diseases.

**Figure 1:**
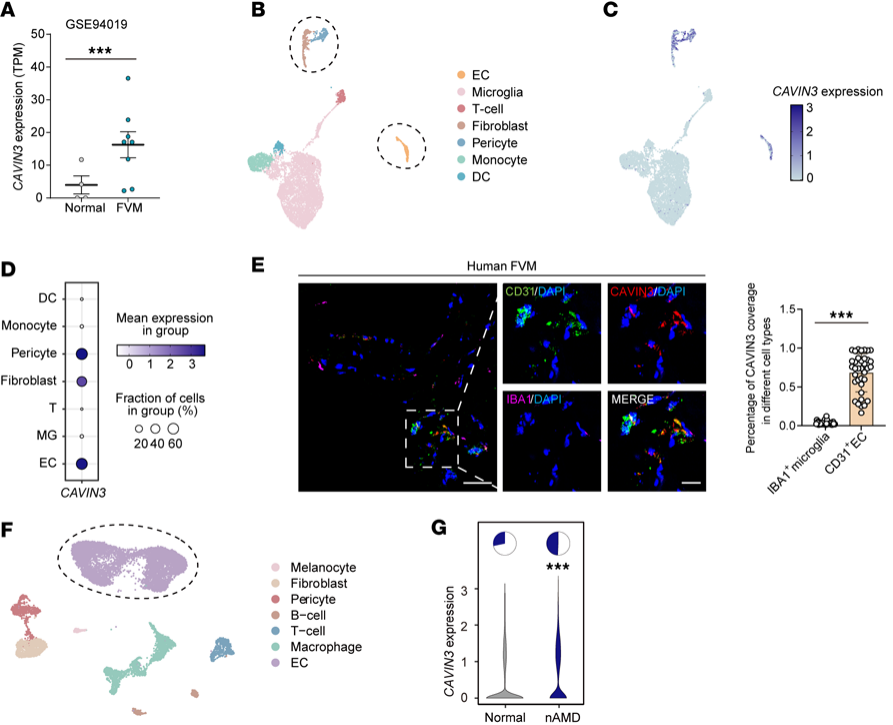
CAVIN3 is upregulated in clinical ocular neovascular diseases. (**A**) RNA-seq data were obtained from a published dataset of FVMs from patients with PDR (GSE94019). *CAVIN3* expression was analyzed in FVMs from PDR patients (*n* = 8) and normal humans (*n* = 4). (**B**) UMAP visualization of scRNA-seq data (GSE165784) from FVMs of patients with PDR. (**C** and **D**) Dot plot (**C**) and bubble plot (**D**) showing *CAVIN3* expression across distinct cell types of FVMs. (**E**) Immunofluorescent staining (CAVIN3, CD31, and IBA1) in FVMs obtained from PDR patients. Scale bars: 20 μm. *n* = 36 samples. (**F**) The UMAP plot displays ECs derived from publicly available CD31-enriched scRNA-seq data from patients with nAMD (GSE135922). (**G**) The violin plot illustrating the relative expression of *CAVIN3* in patients with nAMD compared to the control group. The pie chart indicates the percentage of *CAVIN3*-positive cells. TPM, transcripts per kilobase million. Data are presented as mean ± SD. ***P < 0.001 by 2-tailed Student’s *t* test (**A** and **E**) or Wilcoxon’s rank-sum test (**G**).

CAVIN3 expression in the retina and choroid of patients with PDR and nAMD was first analyzed to confirm its potential role in clinical ocular neovascular disease. The formation of vitreous fibrovascular membranes (FVMs) is a typical pathological feature of PDR, and these membranes can comprise a diverse range of cells, including vascular ECs (22). We reanalyzed previously reported RNA-seq data (NCBI GEO GSE94019) (23) and found elevated expression levels of *CAVIN3* in ECs isolated from FVMs in patients with PDR (Figure 1A). To further investigate the distribution of cell types expressing CAVIN3 in PDR, we analyzed publicly available single-cell RNA-seq (scRNA-seq) data from FVMs of patients with PDR (GEO GSE165784) (24) (Figure 1B). Our analysis revealed a marked enrichment of *CAVIN3* expression in vascular-related cells (Figure 1, C and D). Immunofluorescent staining demonstrated that CAVIN3 colocalized with ECs marked by platelet EC adhesion molecule-1 (CD31) in FVMs of patients with PDR (Figure 1E). Although scRNA-seq analyses from previous studies indicated that microglia were the predominant cell type in FVMs (24), CAVIN3 showed minimal colocalization with microglia labeled by ionized calcium–binding adaptor molecule 1 (IBA1) (Figure 1E). Quantitative analysis revealed that CAVIN3 was expressed in approximately 68% of ECs (Figure 1E). This finding further confirms the association of endothelial CAVIN3 with pathological neoangiogenic processes.

Subsequently, we reanalyzed publicly available scRNA-seq data (GEO GSE135922) (25) of the retinal pigment epithelium (RPE)/choroidal complex in nAMD patients, extracting the CD31-labeled EC population for subsequent evaluation (Figure 1F). We found that *CAVIN3* expression was upregulated in the ECs of patients with nAMD (Figure 1G). In conclusion, these results indicate that CAVIN3 expression in ECs is upregulated in clinical ocular neovascular diseases, suggesting a potential role in the generation and development of pathological neovascularization.

### Cavin3 is upregulated in ocular neovascularization models.

**Figure 2:**
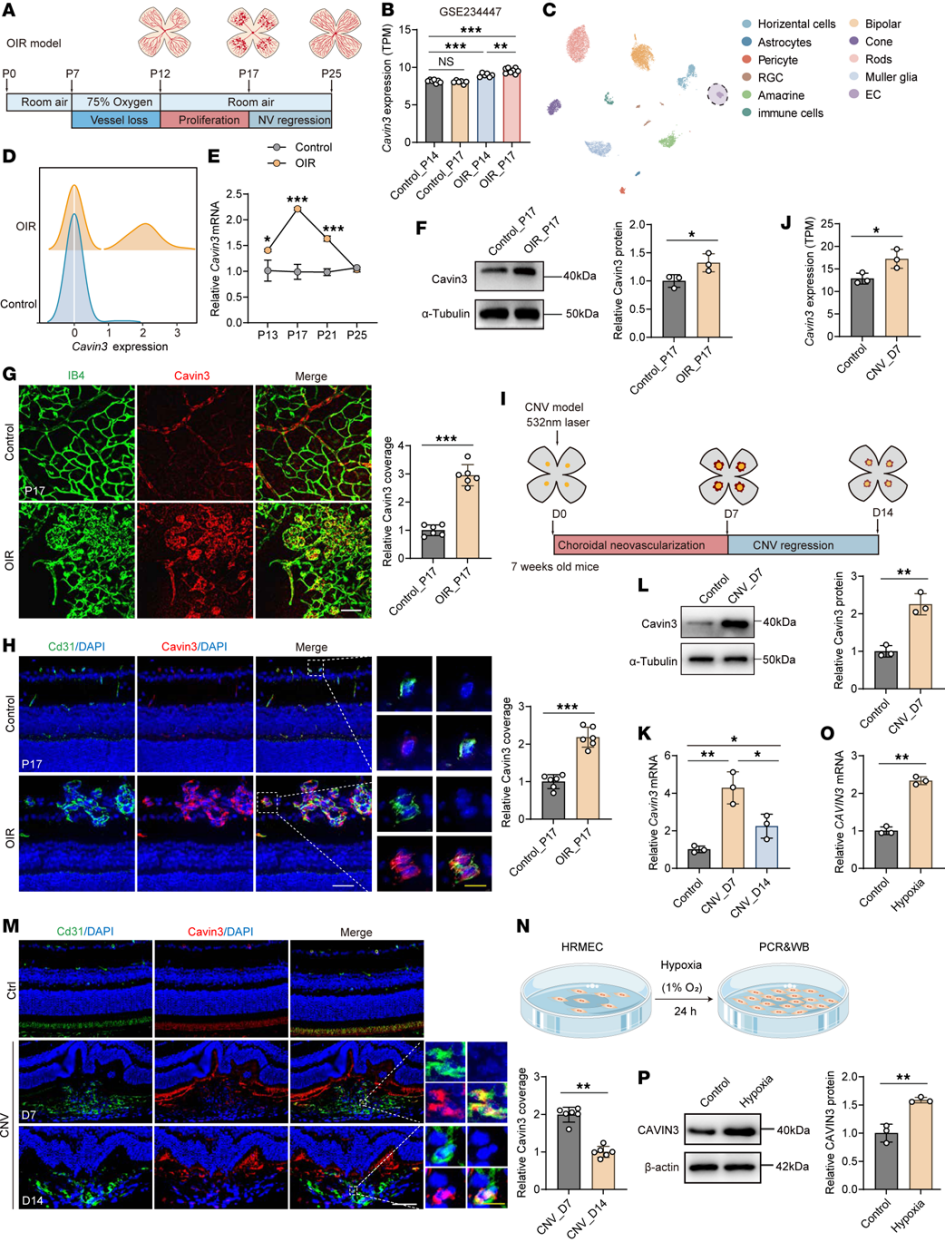
Cavin3 is upregulated in ocular neovascularization models. (**A**) Experimental scheme of OIR. (**B**) Differential *Cavin3* expression in retinas of Control_P14 (*n* = 7), Control_P17 (*n* = 6), OIR_P14 (*n* = 6), and OIR_P17 (*n* = 8) mice (GSE234447). (**C**) UMAP visualization of scRNA-seq data (GSE150703). (**D**) A ridge plot shows *Cavin3* expression in ECs. (**E**) *Cavin3* mRNA in control and OIR retinas on P13, P17, P21, and P25. *n* = 3 per group. (**F**) Cavin3 protein in control and P17 OIR retinas, with α-tubulin as an internal reference. *n* = 3 per group. (**G**) Immunofluorescent staining of IB4 and Cavin3 in retinal flat mounts of control and P17 OIR mice. *n* = 6 per group. Scale bar: 50 μm. (**H**) Immunofluorescent staining of Cd31 and Cavin3 in retinal cryosections of control and OIR mice. *n* = 6 per group. Scale bars: 50 μm (white) and 10 μm (yellow). (**I**) Experimental scheme of CNV. (**J**) *Cavin3* expression in control and CNV_D7 mice from bulk RNA-seq data (GSE207171). *n* = 3 per group. (**K**) mRNA levels of *Cavin3* in the RPE-choroid-sclera complex of control, CNV_D7, and CNV_D14 mice. *n* = 3 per group. (**L**) Immunoblotting of Cavin3 in the RPE-choroid-sclera complex of control and CNV_D7, using α-tubulin as an internal reference. *n* = 3 per group. (**M**) Immunofluorescent costaining of Cd31 and Cavin3 in frozen sections of eyes from control, CNV_D7, and CNV_D14 mice. *n* = 6 per group. Scale bars: 50 μm (white) and 10 μm (yellow). (**N**) Experimental protocols for hypoxic treatment. (**O** and **P**) mRNA (**O**) and protein (**P**) levels of CAVIN3 in HRMECs after control or hypoxic treatment. β-Actin was used as an internal reference. *n* = 3 per group. Data are presented as mean ± SD. *P < 0.05; **P < 0.01; ***P < 0.001 by 1-way ANOVA with Tukey’s multiple-comparison test (**B** and **K**) or 2-tailed Student’s *t* test (**E**–**H**, **J**, **L**, **M**, **O**, and **P**).

To investigate the role of Cavin3 further, we confirmed whether Cavin3 expression in mouse neovascular models is consistent with clinical ocular neovascular diseases. We first utilized the OIR mouse model to assess Cavin3 expression in retinopathy (Figure 2A). The OIR model produces retinal neovascularization and avascular perfusion zones similar to those observed in PDR and ROP, with neovascularization peaking on P17 (26). We first assessed the expression of Cavin3 in the whole retina of OIR by reanalyzing bulk RNA-seq data (GEO GSE234447) (27) from OIR and control mouse retinas. *Cavin3* expression was found to be upregulated in OIR through differentially expressed gene (DEG) analysis, with the expression on P17 (the peak of retinal neovascularization) higher than on P14 (Figure 2B and Supplemental Figure 1, A and B; supplemental material available online with this article; https://doi.org/10.1172/jci.insight.187836DS1). To further evaluate the endothelial Cavin3 expression pattern in OIR retinas, we reanalyzed scRNA-seq (GEO GSE150703) (28) data from OIR at P17. The scRNA-seq data were visualized using uniform manifold approximation and projection (UMAP) for retinal cell types, with classical markers employed to identify subpopulations (Figure 2C and Supplemental Figure 1C). Retinal ECs were extracted from the scRNA-seq objects for DEG analysis, identifying *Cavin3* upregulation in OIR (Figure 2D and Supplemental Figure 1, D and E). In the joint analysis of upregulated DEGs from scRNA-seq and bulk RNA-seq data, *Cavin3* emerged as a shared upregulated DEG, further confirming its potential pathological role in ECs during OIR progression (Supplemental Figure 1F). To further assess the expression of Cavin3, we examined the mRNA levels of retinal *Cavin3* in OIR and control mice at multiple time points (P13, P17, P21, and P25). Consistent with the sequencing results, *Cavin3* mRNA levels were increased in OIR retinas, peaking at the time of maximal neovascularization (P17) (Figure 2E). Immunoblotting indicated that Cavin3 protein levels were elevated in the retinas of OIR mice on P17 compared with control mice (Figure 2F). To further explore the role of Cavin3, we examined its expression in the ECs of mouse retina. Immunofluorescent staining of retinal flat mounts from P17 mice revealed that Cavin3 was expressed exclusively in ECs (stained with isolectin-B4 [IB4]) in the mouse retina and showed an increased expression in neovascular clusters in the OIR retinas (Figure 2G). We also stained frozen sections of eyes from P17 mice and found that Cavin3 predominantly colocalized with Cd31, indicating its EC-specific distribution (Supplemental Figure 2). However, this colocalization was absent for Cavin3 in Iba1-labeled microglia (Supplemental Figure 2). Further analysis of the costaining results for Cd31 and Cavin3 revealed that Cavin3 expression was elevated in Cd31-labeled ECs in OIR (Figure 2H). Collectively, these results confirm the potential role of Cavin3 in EC involvement in OIR angiogenesis.

To further investigate the expression pattern of endothelial Cavin3 in ocular neovascularization, we next examined Cavin3 expression in the CNV mouse model, which mimics the conditions of nAMD (29). Following laser-induced damage to Bruch’s membrane, new vessels grow from the choroid toward the retina (30). Notably, CNV began to regress spontaneously by the seventh day (Figure 2I). Upon reanalyzing bulk RNA-seq (GEO GSE207171) (31) data from the CNV model, we found that *Cavin3* was upregulated in CNV (Figure 2J). Quantitative PCR (qPCR) results indicated that the mRNA level of *Cavin3* was elevated in CNV compared with controls, with higher expression observed on day 7 (CNV_D7) (the peak of CNV) compared with day 14 (CNV_D14) (Figure 2K) (32). Immunoblotting results indicated that the protein level of Cavin3 in the RPE-choroid-sclera complex of CNV mice was higher than that in controls (Figure 2L). In immunofluorescent staining of frozen sections from control and CNV mice, we compared Cavin3 expression in the ECs. Immunofluorescent costaining for Cavin3 and Cd31 in vascular ECs revealed higher Cavin3 expression in ECs on day 7 compared with day 14 (Figure 2M).

Hypoxia is a major inducer of microvascular injury (33). Increasing evidence suggests that hypoxia-stimulated retinal vascular EC dysfunction is the pathological basis for the development of ocular neovascular disease (34). To further investigate the effect of CAVIN3 on the function of vascular ECs in pathological status, we exposed human retinal microvascular ECs (HRMECs) to hypoxia in vitro and assessed CAVIN3 expression (Figure 2N). We confirmed that CAVIN3 mRNA and protein levels increased in HRMECs under hypoxic treatment (Figure 2, O and P).

In conclusion, these findings suggest an association between endothelial Cavin3 expression and ocular neovascularization, particularly during the peak of neovascularization.

### CAVIN3 deficiency prevents pathological neovascularization.

**Figure 3:**
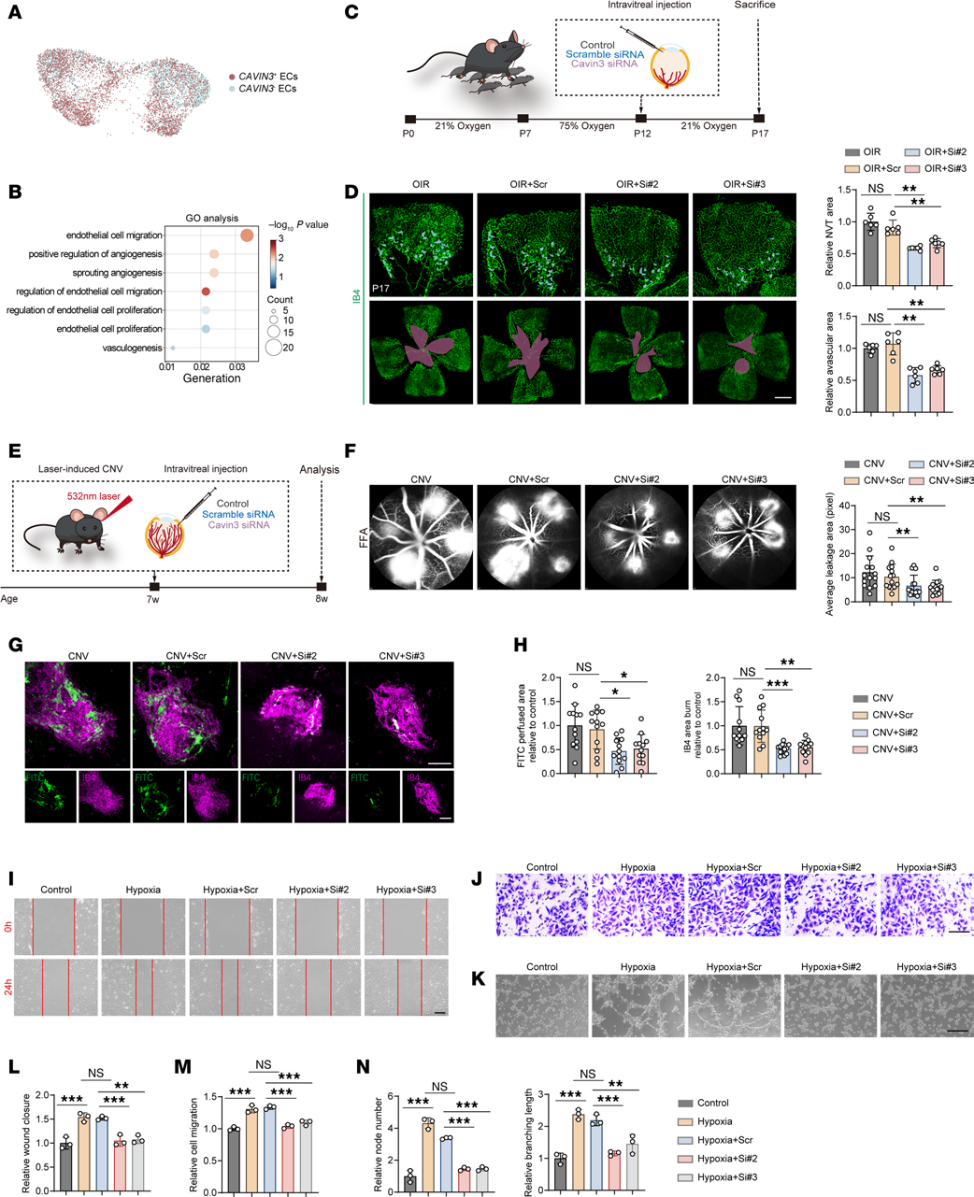
CAVIN3 deficiency prevents pathological neovascularization. (**A**) *CAVIN3^+^* ECs (red dots) and *CAVIN3^–^* ECs (blue dots) are shown on the UMAP plot. (**B**) GO analysis reveals biological processes underlying the major enrichment of DEGs between *CAVIN3^+^* ECs and *CAVIN3^–^* ECs. (**C**) Experimental scheme for **D** and Figure 4. (**D**) Immunofluorescent staining of IB4 in retinas from OIR mice without treatment or OIR mice injected with scramble siRNA/Cavin3-siRNA on P17. Blue areas indicate NVTs in the upper, partially magnified retinal flat mounts, while magenta areas mark avascular areas. *n* = 6 per group. Scale bar: 50 μm. (**E**) Experimental protocols for **F** and **G**. (**F**) Fluorescein fundus angiography (FFA) images of CNV mice without treatment or CNV mice injected with scramble siRNA/Cavin3-siRNA. CNV leakage area was quantified and compared. *n* = 14 burns. (**G** and **H**) Immunofluorescent staining of IB4 and FITC-dextran in the RPE-choroid-sclera complex of CNV mice without treatment or CNV mice injected with scramble siRNA/Cavin3-siRNA. *n* = 12 burns per group. Scale bars: 100 μm. (**I** and **L**) Scratch test on untreated HRMECs, hypoxia-treated HRMECs, or hypoxia-treated HRMECs transfected with scramble siRNA/CAVIN3-siRNA. *n* = 3 per group. Scale bar: 200 μm. (**J** and **M**) Transwell migration assay on untreated HRMECs, hypoxia-treated HRMECs, or hypoxia-treated HRMECs transfected with scramble siRNA/CAVIN3-siRNA. *n* = 3 per group. Scale bar: 100 μm. (**K** and **N**) Tube formation assay on untreated HRMECs, hypoxia-treated HRMECs, or hypoxia-treated HRMECs transfected with scramble siRNA/CAVIN3-siRNA. *n* = 3 per group. Scale bars: 100 μm. Data are presented as mean ± SD. *P < 0.05; **P < 0.01; ***P < 0.001 by 1-way ANOVA with Tukey’s multiple-comparison test.

Based on the scRNA-seq data obtained from patients with nAMD (GEO GSE135922) (25), we clustered *CAVIN3*^+^ ECs and *CAVIN3*^–^ ECs and performed DEG analysis (Figure 3A). Gene Ontology (GO) enrichment analysis of the DEGs revealed that they were mainly associated with biological processes related to angiogenesis, vascular sprouting, and vasculogenesis (Figure 3B), suggesting a role for CAVIN3 in the development of angiogenesis.

Next, we examined whether angiogenesis in OIR and CNV mouse models was affected by Cavin3. Since the protein sequence of Cavin3 is highly conserved between rats, humans, and mice (Supplemental Figure 3), we designed 4 siRNAs targeting the *Cavin3* gene for in vivo use in mice. We selected 2 of the most efficient siRNAs, Cavin3-siRNA2 (Si2) and Cavin3-siRNA3 (Si3) (Supplemental Figure 4, A and B), for vitreous cavity injection to modulate Cavin3 expression. Retinal flat mount staining revealed that the expression of Cavin3 in Cd31-labeled ECs was markedly reduced in the Cavin3-siRNA–treated group (Supplemental Figure 4C). We injected Cavin3-siRNA into the vitreous cavity of OIR mice on P12, which is the onset of retinal neovascularization and vascular leakage in OIR models, with P17 considered the peak period for lesion angiogenesis (35). On P17, retinas were collected and analyzed (Figure 3C). A substantial reduction in the area of neovascular tufts (NVTs) and the avascular zone in the central retina was observed in OIR mice receiving Cavin3-siRNA injections compared with OIR mice without injection or those injected with scramble siRNA (Scr) (Figure 3D).

The effects of Cavin3 on the choroid were further investigated using a CNV mouse model (Figure 3E). Fluorescein fundus angiography revealed a marked reduction in CNV leakage area in Cavin3-knockdown mice (Figure 3F). To quantify the CNV area, IB4 staining and FITC-dextran were used to visualize the neovasculature. The CNV area, as indicated by IB4 and FITC-dextran fluorescence, was reduced in mice injected with Cavin3-siRNA (Figure 3, G and H).

To further elucidate the role of CAVIN3 in blocking pathological angiogenesis, we designed and selected 2 distinct siRNA sequences with the highest knockdown efficiency, CAVIN3-siRNA2 (Si2) and CAVIN3-siRNA3 (Si3) (Supplemental Figure 5, A and B), to regulate CAVIN3 expression in HRMECs. Scratch (Figure 3, I and L) and Transwell migration (Figure 3, J and M) assays demonstrated that the migratory ability of CAVIN3-siRNA–treated HRMECs was reduced, indicating that CAVIN3 knockdown inhibited hypoxia-induced EC migration. Additionally, tube formation experiments showed that tube formation was inhibited in HRMECs from the CAVIN3-knockdown group under hypoxic conditions, as assessed by analyzing the number of nodes and branch length (Figure 3, K and N). All these results demonstrate that CAVIN3 deficiency inhibits hypoxia-promoted EC migration and tube-forming ability in vitro. Taken together, these findings indicate that Cavin3 deficiency inhibits pathological neovascularization.

### Cavin3 deficiency promotes vascular normalization by partially restoring microenvironmental hypoxia and reestablishing pericyte-EC interactions.

**Figure 4:**
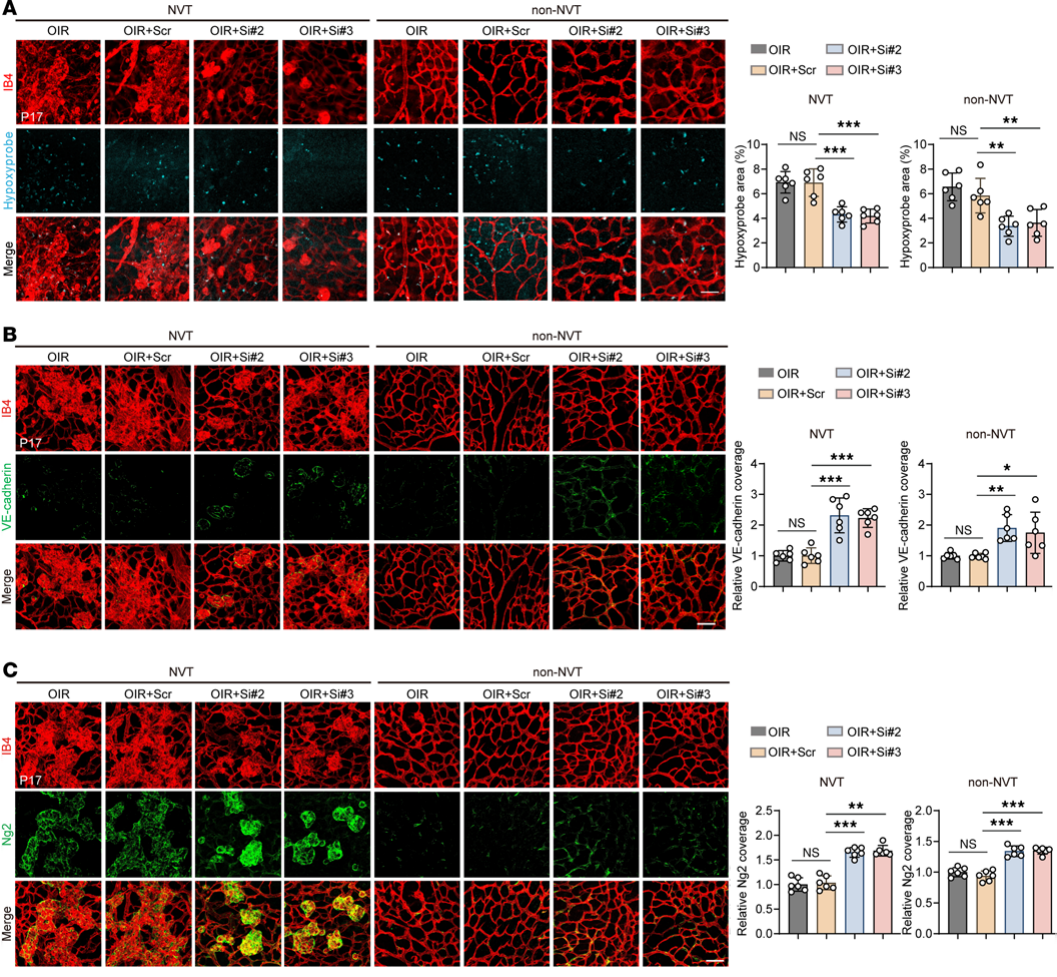
Cavin3 deficiency promotes vascular normalization by partially restoring microenvironmental hypoxia and reestablishing pericyte-EC interactions. (**A**) Hypoxyprobe and IB4 immunofluorescent staining was conducted in the NVT and non-NVT areas of retinal flat mounts from OIR mice without treatment or OIR mice injected with scramble siRNA/Cavin3-siRNA on P17. *n* = 6 per group. Scale bar: 50 μm. (**B**) Immunofluorescent staining for VE-cadherin and IB4 in NVT and non-NVT areas of retinal flat mounts from OIR mice without treatment or OIR mice injected with scramble siRNA/Cavin3-siRNA on P17. *n* = 6 per group. Scale bar: 50 μm. (**C**) Immunofluorescent staining for Ng2 and IB4 in NVT and non-NVT areas of retinal flat mounts from OIR mice without treatment or OIR mice injected with scramble siRNA/Cavin3-siRNA on P17. *n* = 6 per group. Scale bar: 50 μm. Data are presented as mean ± SD. *P < 0.05; **P < 0.01; ***P < 0.001 by 1-way ANOVA with Tukey’s multiple-comparison test.

The formation of a well-functioning vascular network is essential for the survival and proper functioning of organs (36). Key features of dysfunctional vessels include disrupted pericyte-EC interactions and the presence of hypoxia (34, 37). Hypoxia, in turn, drives the growth of abnormal vessels (33). Given that Cavin3 knockdown has an inhibitory effect on ocular pathological angiogenesis, we next investigated the effect of Cavin3 deficiency in vascular normalization. Retinal microenvironmental hypoxia is known to be the primary driver of neovascularization and development in OIR, so we assessed the hypoxic status of the OIR retina using hypoxyprobe. The results indicated that the hypoxic signaling was downregulated in the NVT region after Cavin3 knockdown, as well as in the surrounding non-NVT region, as demonstrated by hypoxyprobe and IB4 immunofluorescently stained retinal flat mounts (Figure 4A). This finding suggests that Cavin3 knockdown alleviates the hypoxic state of the retinal microenvironment, thereby reducing the factors that induce neovascularization.

Normal vessels are lined with a quiescent monolayer of adherent ECs, which are interconnected, polarized, and aligned in the direction of blood flow to ensure optimal perfusion (38). In contrast, pathological ECs are often leaky and exhibit disturbed junctions, leading to hemorrhage and increased interstitial fluid pressure, which restricts perfusion (39–41). Vascular endothelial cadherin (VE-cadherin) is essential for maintaining the structural and functional integrity of the EC barrier and inhibiting angiogenesis to some extent (42). Staining of flat mounts of OIR retinas revealed that the area of VE-cadherin coverage was restored in both NVTs and non-NVT regions of OIR retinas following Cavin3 knockdown (Figure 4B). In contrast, discontinuous VE-cadherin fluorescence was observed in the OIR retinas and those injected with scramble siRNA (Figure 4B).

The role of pericytes as stabilizers of maturing vessels is well established (15). They are located around EC junctions, forming umbrella-like structures that cover gaps between ECs and regulate barrier function (43). In pathological vessels, pericyte abnormalities contribute to flow disruptions (44, 45). Abnormal pericytes in these vessels are loosely associated with ECs, and pericyte coverage is typically reduced (46, 47). We utilized the pericyte marker neuroglial antigen 2 (Ng2) for immunofluorescent staining of OIR retinal flat mounts to evaluate the effect of Cavin3 deficiency on vascular EC-pericyte interactions in the mouse retina. Our analysis revealed that pericyte coverage in both NVTs and non-NVT regions was increased in Cavin3-siRNA–treated OIR retinas (Figure 4C).

These results indicate that Cavin3 deficiency promotes vascular normalization by partially restoring microenvironmental hypoxia and reestablishing pericyte-EC interactions.

### CAVIN3 deficiency inhibits EC proliferation in pathological conditions.

**Figure 5:**
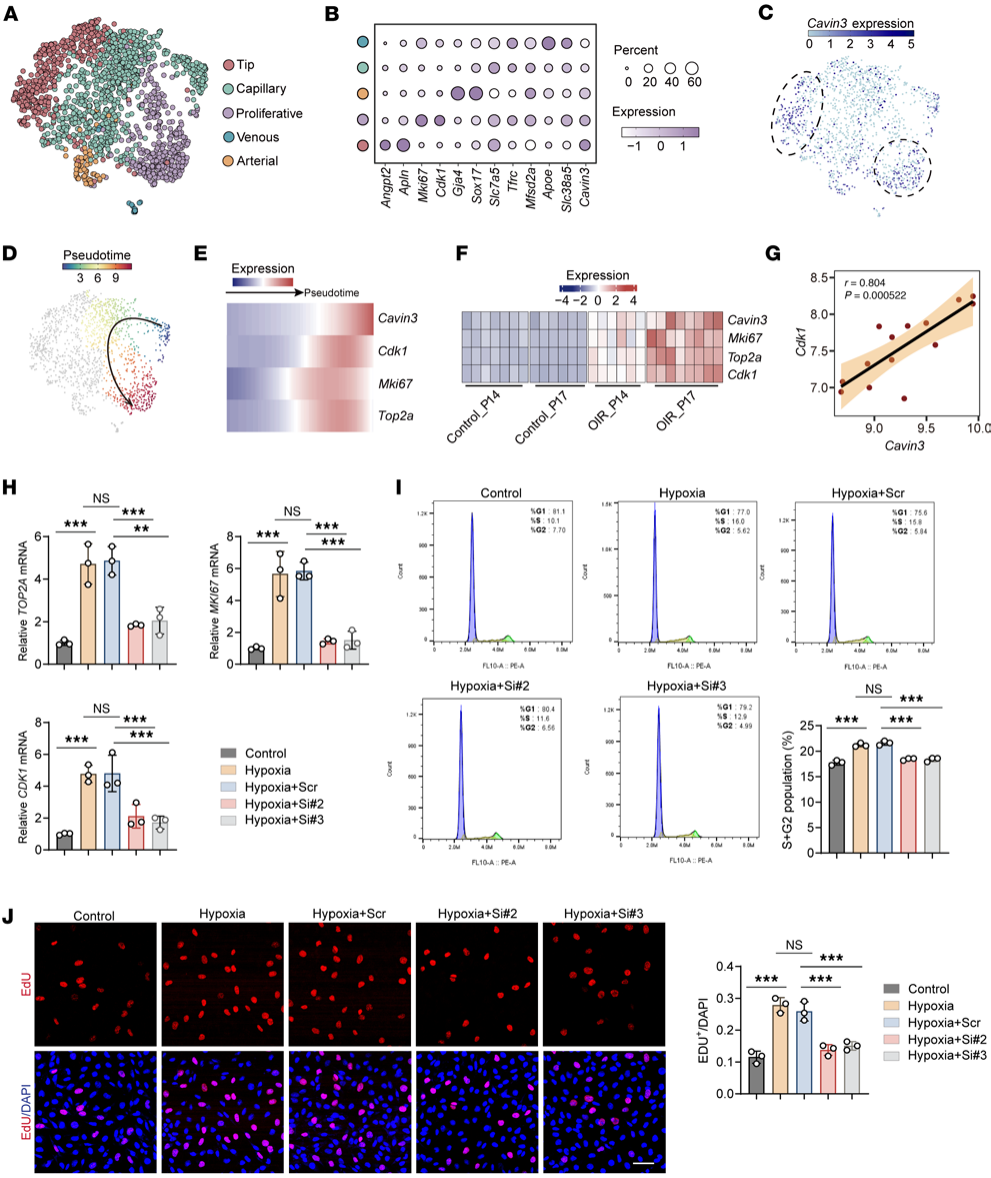
CAVIN3 deficiency inhibits EC proliferation in pathological conditions. (**A**) UMAP visualization plot classifying subpopulations of Cd31-positive cells in the retinas of OIR mice, where ECs are categorized as tip, proliferative, capillary, arterial, and venous ECs. (**B**) Bubble plot displays the percentage and expression of classical marker–positive cells in each subpopulation. (**C**) Dot plot illustrates the distribution of *Cavin3* in the clusters of retinal ECs. (**D** and **E**) Pseudotime-ordered analyses show the expression of *Cavin3* and proliferative markers during the differentiation of common ECs into proliferative ECs. (**F**) A heatmap depicts the changes in *Cavin3* and proliferative markers along with the pseudotime. (**G**) Correlation analysis shows a correlation between *Cavin3* and *Cdk1* expression. (**H**) mRNA levels of proliferation marker genes (*TOP2A*, *MKI67*, and *CDK1*) in control HRMECs, hypoxia-treated HRMECs, or hypoxia-treated HRMECs transfected with scramble siRNA/CAVIN3-siRNA. *n* = 3 per group. (**I**) Flow cytometry analysis of control HRMECs, hypoxia-treated HRMECs, or hypoxia-treated HRMECs transfected with scramble siRNA/CAVIN3-siRNA. *n* = 3 per group. (**J**) EDU assay on control HRMECs, hypoxia-treated HRMECs, or hypoxia-treated HRMECs transfected with scramble siRNA/CAVIN3-siRNA. *n* = 3 per group. Scale bar: 50 μm. Data are presented as mean ± SD. **P < 0.01, ***P < 0.001 by 1-way ANOVA with Tukey’s multiple-comparison test (**H**–**J**).

We next aimed to identify the specific pathogenic process mediated by EC Cavin3 deficiency that leads to vascular normalization in mouse retinas. Using UMAP, we classified and visualized ECs in the scRNA-seq (GEO GSE174400) (48) of OIR retina, categorizing them as tip cells, capillaries, proliferating ECs, venous ECs, and arterial ECs (Figure 5A). We found that *Cavin3* in ECs was primarily concentrated in proliferative cells and tip cells (Figure 5, B and C). In the advanced proliferative stages of PDR and ROP, NVTs proliferate on the retinal surface, exacerbating complications such as retinal detachment and ultimately leading to blindness (47, 49). We further evaluated the role of Cavin3 in regulating the proliferative capacity of pathological neovascular ECs. Previous literature has shown that Cdk1, Mki67, and Top2a serve as markers of cell proliferation (50–52). In the pseudotime-ordered analyses, we observed that *Cavin3* expression was upregulated as normal ECs differentiated into proliferative states, alongside the upregulation of classic proliferation markers (Figure 5, D and E). Consistently, analysis of bulk RNA-seq (GEO GSE234447) (27) data from OIR mice revealed that *Cavin3* levels increased alongside markers of proliferative cells over time, as shown in Figure 2B in the retina of OIR mice (Figure 5F). Additionally, correlation analysis indicated that *Cavin3* expression was positively correlated with the proliferation marker *Cdk1* (Figure 5G). We further investigated the distribution of Cavin3 expression in the vasculature of the central nervous system (CNS). By reanalyzing scRNA-seq data from ECs in the developing brain (GEO GSE111839) (53), we found that *Cavin3* was highly expressed in tip cells and proliferating ECs (Supplemental Figure 6, A–C) and coexpressed with markers indicative of proliferative properties (Supplemental Figure 6, D–F). This suggests that CAVIN3 is a potential gene involved in the general regulation of EC proliferation.

To confirm the regulatory role of CAVIN3 in EC proliferation, we performed CAVIN3 knockdown in hypoxia-induced HRMECs. We found that the mRNA levels of proliferation-associated markers were reduced in CAVIN3-knockdown HRMECs following hypoxic treatment (Figure 5H). Flow cytometry analysis confirmed that under hypoxic conditions, the number of S-phase and G_2_/M-phase cells in CAVIN3-siRNA–transfected HRMECs decreased, leading to cell cycle arrest in the G_1_ phase compared with the scramble siRNA group (Figure 5I). Additionally, 5-ethynyl-2′-deoxypyridine (EdU) experiments further confirmed that CAVIN3 knockdown inhibited hypoxia-mediated cell proliferation in HRMECs (Figure 5J). All these results indicate that CAVIN3 deficiency prevents hypoxia-promoted EC proliferative capacity.

### Cavin3 deficiency inhibits pathological vascular sprouting.

**Figure 6:**
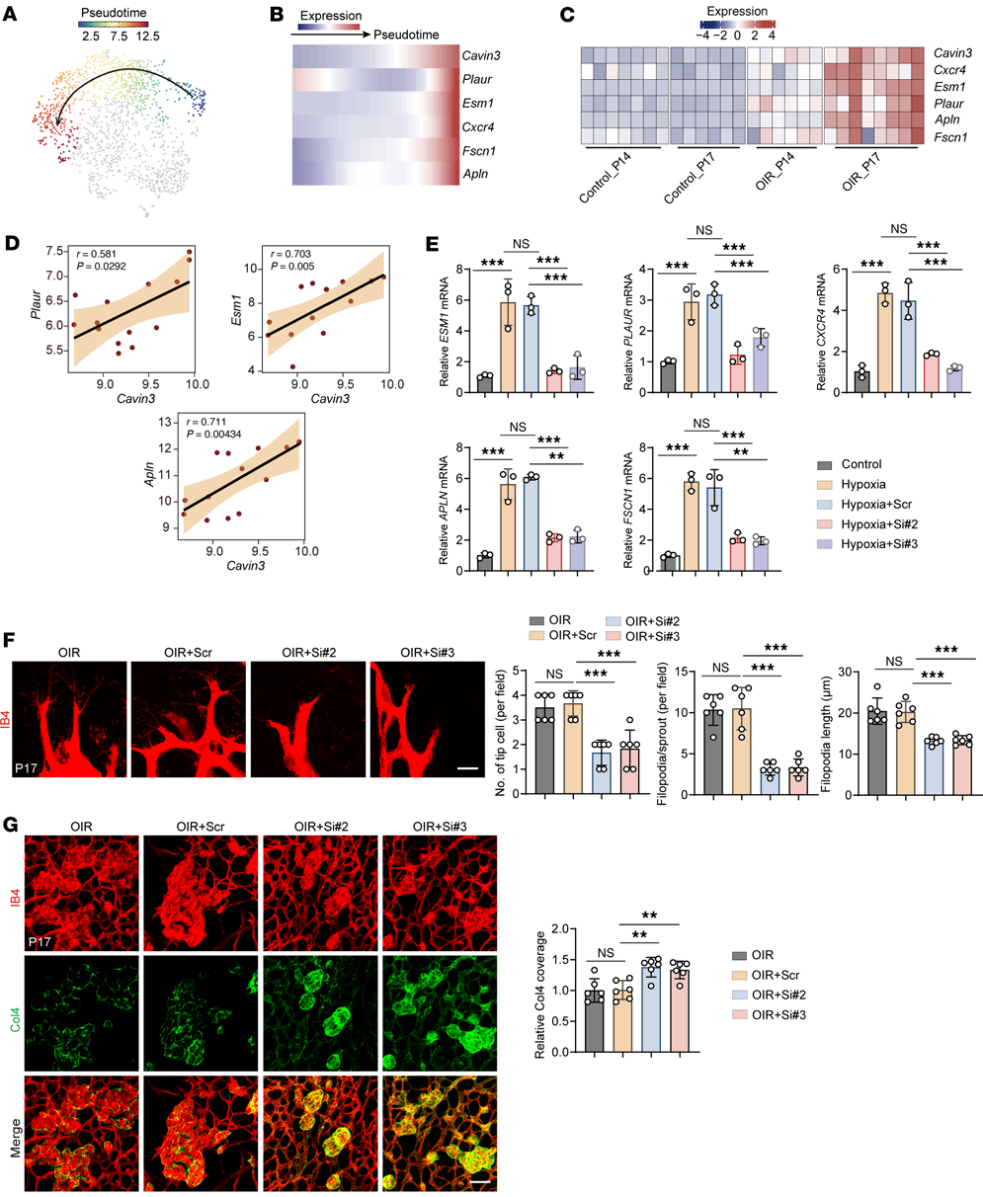
Cavin3 deficiency inhibits pathological vascular sprouting. (**A** and **B**) Pseudotime-ordered analyses show the expression of *Cavin3* and marker genes associated with tip cells during the differentiation of common ECs into tip cells. (**C**) A heatmap depicts the changes in *Cavin3* and marker genes for tip cells over time in the retinas of OIR mice. (**D**) Correlation analyses show a positive correlation between *Cavin3* and tip cell markers (*Plaur*, *Esm1*, and *Apln*) expression. (**E**) mRNA levels of tip cell marker genes (*ESM1*, *PLAUR*, *CXCR4*, *APLN*, and *FSCN1*) in untreated HRMECs, hypoxia-treated HRMECs, or hypoxia-treated HRMECs transfected with scramble siRNA/CAVIN3-siRNA. *n* = 3 per group. (**F**) High-resolution images of IB4-stained retinas show tip cells at the angiogenic front of OIR mice without treatment or OIR mice injected with scramble siRNA/Cavin3-siRNA on P17. *n* = 6 per group. Scale bar: 10 μm. (**G**) Col4 and IB4 immunofluorescent staining of the NVT region in retinal flat mounts from OIR mice without treatment or OIR mice injected with scramble siRNA/Cavin3-siRNA on P17. *n* = 6 per group. Scale bar: 50 μm. Data are presented as mean ± SD. **P < 0.01, ***P < 0.001 by 1-way ANOVA with Tukey’s multiple-comparison test (**E**–**G**).

In addition to proliferating ECs, Cavin3 was also found to be highly expressed in tip cells (Figure 5, B and C). The tip cells respond to guidance cues presented by the neighboring tissues and various cell types to facilitate angiogenic sprouting (54). To assess the regulatory role of Cavin3 in pathological vascular sprouting, we performed a pseudotime-ordered analysis of ECs derived from the scRNA-seq (GEO GSE174400) (48) of OIR. This analysis revealed that the expression of *Cavin3* was upregulated as normal ECs differentiated into tip cells (Figure 6, A and B). Additionally, *Cavin3* expression was found to be elevated alongside tip cell markers over time, with correlation analyses showing a proportional relationship with the expression of multiple tip cell markers (Figure 6, C and D). In the immunofluorescent staining of retinal flat mounts from P4 mice, we observed that Cavin3 was primarily enriched in the tip cells in the angiogenic front of the developing retina (Supplemental Figure 7), consistent with the results of our scRNA-seq analyses. Furthermore, *Cavin3* was highly expressed in CNS tip cells (Supplemental Figure 6, A–C) and coexpressed with tip markers (Supplemental Figure 6, G–K) in CNS vessels. These results suggest a potential role for Cavin3 in promoting endothelial tip cell formation.

Under hypoxic treatment, mRNA levels of tip cell markers were reduced in CAVIN3-knockdown HRMECs compared with the scramble siRNA group (Figure 6E). This result demonstrates that CAVIN3 deficiency inhibits hypoxia-promoted EC-to–tip cell formation ability in vitro.

We further verified the inhibitory effect of Cavin3 deficiency on pathological vessel sprouting in OIR mice. By analyzing the tip cells at the anterior end of the NVTs, we found that the number of tip cells, sprouted filopodia, and the length of filopodia were reduced in Cavin3-knockdown mice (Figure 6F). Furthermore, during the sprouting process, the extracellular matrix (ECM) provides a crucial scaffold for EC attachment and migration and modulates signal transduction pathways essential for EC morphogenesis (55). Local protein hydrolysis of collagen type IV (Col4), a marker of the ECM, is required for vascular EC germination (56). Therefore, we assessed Col4 retinal vascular coverage in the NVT region of the OIR retina. An increase in Col4 coverage in the NVT region was observed in the Cavin3-knockdown group (Figure 6G). Collectively, we conclude that Cavin3 deficiency inhibits the sprouting of pathological blood vessels and promotes their vascular normalization process.

### ERK phosphorylation is essential for CAVIN3-mediated regulation of pathological angiogenesis.

**Figure 7:**
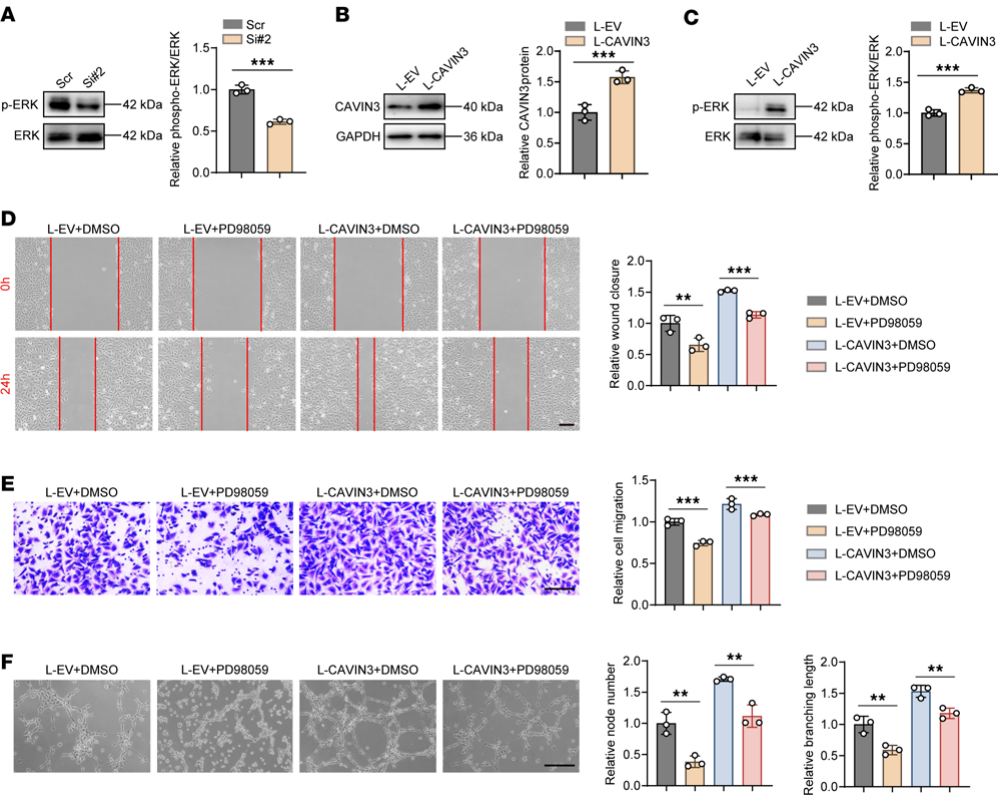
ERK phosphorylation is essential for CAVIN3-mediated regulation of pathological angiogenesis. (**A**) Immunoblotting of ERK and p-ERK in HRMECs following scramble siRNA/CAVIN3-siRNA treatment, using GAPDH as an internal reference. *n* = 3 per group. (**B**) Immunoblotting of CAVIN3 in HRMECs transduced with L-EV/L-CAVIN3, using GAPDH as an internal reference. *n* = 3 per group. (**C**) Immunoblotting of ERK and p-ERK in HRMECs transduced with L-EV/L-CAVIN3, using GAPDH as an internal reference. *n* = 3 per group. (**D**) Scratch test on DMSO-treated HRMECs transduced with L-EV/L-CAVIN3 or PD98059-treated HRMECs transduced with L-EV/L-CAVIN3. *n* = 3 per group. Scale bar: 200 μm. (**E**) Transwell migration assay on DMSO-treated HRMECs transduced with L-EV/L-CAVIN3 or PD98059-treated HRMECs transduced with L-EV/L-CAVIN3. *n* = 3 per group. Scale bar: 100 μm. (**F**) Tube formation assay on DMSO-treated HRMECs transduced with L-EV/L-CAVIN3 or PD98059-treated HRMECs transduced with L-EV/L-CAVIN3. *n* = 3 per group. Scale bar: 100 μm. Data are presented as mean ± SD. **P < 0.01, ***P < 0.001 by 2-tailed Student’s *t* test (**A**–**C**) or 1-way ANOVA with Tukey’s multiple-comparison test (**D**–**F**).

To further elucidate the molecular mechanisms underlying CAVIN3 function, we validated its downstream targets in HRMECs. Previous studies have demonstrated that CAVIN3 can upregulate ERK signaling by anchoring the caveolae to the cytoskeleton through myosin-1c, while simultaneously downregulating serine/threonine kinase signaling to maintain a balance between the 2 pathways (57). We initially selected ERK as a potential downstream target of CAVIN3 to investigate its pathological role in neovascularization. We found that ERK phosphorylation was inhibited upon CAVIN3 knockdown (Figure 7A). To further verify whether CAVIN3 could regulate ERK phosphorylation, we overexpressed CAVIN3 using a lentivirus (L-CAVIN3) in HRMECs. Immunoblotting revealed the upregulation of both CAVIN3 expression and ERK phosphorylation in HRMECs transduced with L-CAVIN3 (Figure 7, B and C). We then treated these HRMECs with the ERK phosphorylation inhibitor PD98059 (Supplemental Figure 8A). Scratch (Figure 7D) and Transwell migration (Figure 7E) assays confirmed that the migratory capacity of HRMECs induced by CAVIN3 overexpression could be inhibited by PD98059 treatment. Additionally, PD98059 treatment also inhibited the tube-forming ability of HRMECs transduced with L-CAVIN3 (Figure 7F). Collectively, these results confirm that CAVIN3 regulates ERK phosphorylation in the context of pathological angiogenesis.

### CAVIN3 deficiency downregulates JAG1 by inhibiting ERK phosphorylation.

**Figure 8:**
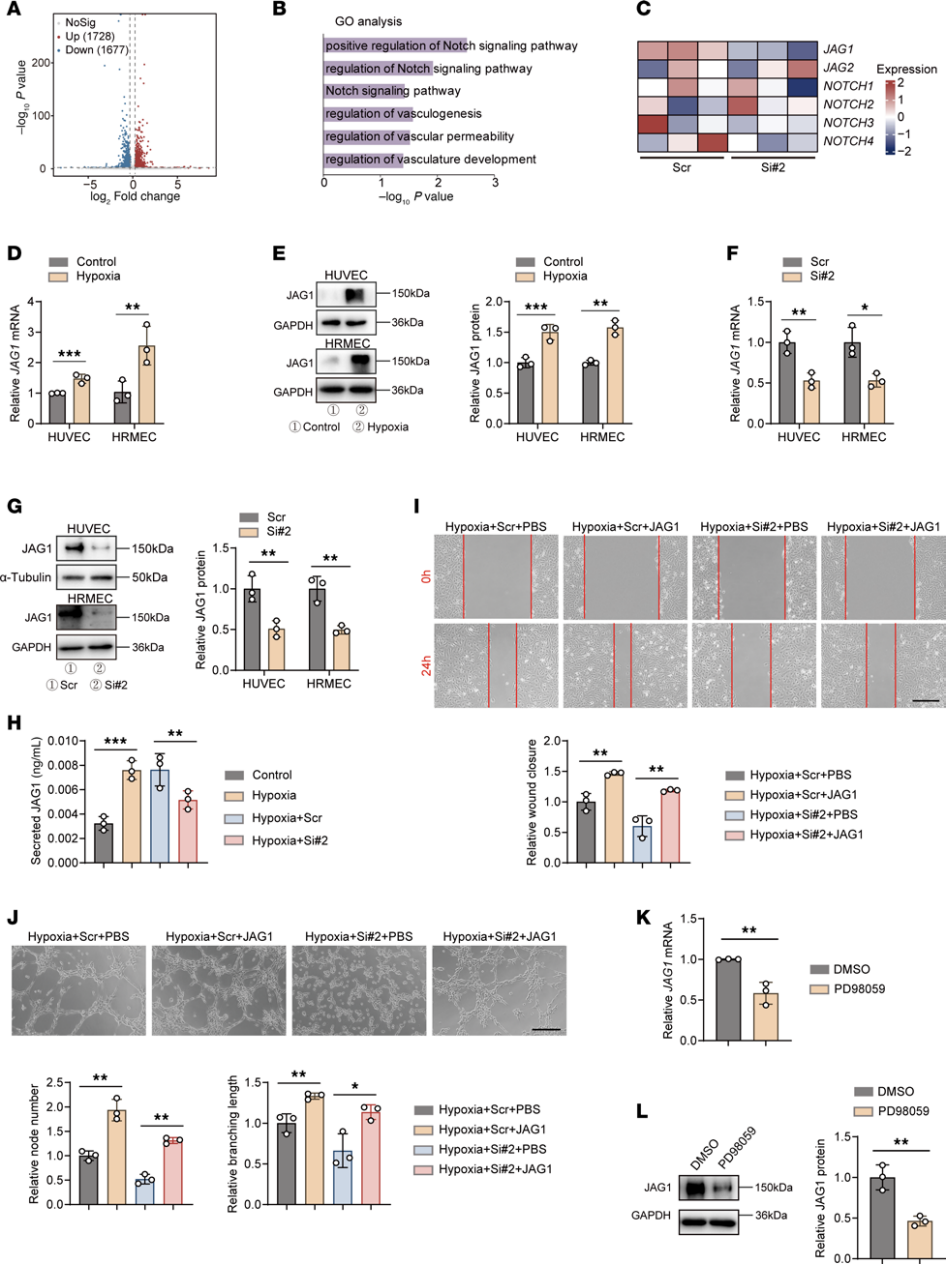
CAVIN3 deficiency downregulates JAG1 by inhibiting ERK phosphorylation. (**A**) A volcano plot displays DEGs of CAVIN3-siRNA–treated HUVECs versus scramble siRNA–treated HUVECs. (**B**) GO analysis revealed the enrichment of significant DEGs in CAVIN3-siRNA–treated HUVECs versus scramble siRNA–treated HUVECs. (**C**) A heatmap illustrates the expression of NOTCH signaling pathway ligand receptors, including *JAG1*, in CAVIN3-siRNA–treated HUVECs versus scramble siRNA–treated HUVECs. (**D**) mRNA levels of *JAG1* in HUVECs and HRMECs after control or hypoxic treatment. *n* = 3 per group. (**E**) Immunoblotting of JAG1 in HUVECs and HRMECs following control or hypoxic treatment, using GAPDH as an internal reference. *n* = 3 per group. (**F**) mRNA levels of *JAG1* in HUVECs and HRMECs transfected with scramble siRNA/CAVIN3-siRNA. *n* = 3 per group. (**G**) Immunoblotting of JAG1 in HUVECs and HRMECs transfected with scramble siRNA/CAVIN3-siRNA. α-Tubulin was used as an internal control in HUVECs, while GAPDH was used in HRMECs. *n* = 3 per group. (**H**) Levels of JAG1 in cell supernatants following control or hypoxic treatment, as well as CAVIN3 knockdown or control treatment of HUVECs. *n* = 3 per group. (**I**) Scratch test on PBS-treated HRMECs transfected with scramble siRNA/CAVIN3-siRNA or JAG1-treated HRMECs transfected with scramble siRNA/CAVIN3-siRNA under hypoxia. *n* = 3 per group. Scale bar: 200 μm. (**J**) Tube formation assay on PBS-treated HRMECs transfected with scramble siRNA/CAVIN3-siRNA or JAG1-treated HRMECs transfected with scramble siRNA/CAVIN3-siRNA under hypoxia. *n* = 3 per group. Scale bar: 100 μm. (**K**) mRNA levels of *JAG1* in HRMECs following PD98059 treatment. *n* = 3 per group. (**L**) Immunoblotting of JAG1 in HRMECs following PD98059 treatment, using GAPDH as an internal reference. *n* = 3 per group. Data are presented as mean ± SD. *P < 0.05; **P < 0.01; ***P < 0.001 by 2-tailed Student’s *t* test (**D**–**G**, **K**, and **L**) or 1-way ANOVA with Tukey’s multiple-comparison test (**H**–**J**).

Given the lack of existing studies on CAVIN3 in clinical diseases, we conducted bulk RNA-seq (GEO GSE275653) to clarify its downstream activation mechanisms. Our research team analyzed samples from human umbilical vein ECs (HUVECs) treated with either scrambled siRNA or CAVIN3-siRNA (Supplemental Figure 5, C and D), identifying a total of 3,405 significant DEGs, including 1,728 upregulated genes and 1,677 downregulated genes (Figure 8A). GO analysis showed that DEGs were primarily enriched in pathways regulating NOTCH signaling and modulating functions such as angiogenesis and permeability (Figure 8B). *JAG1* also appeared markedly upregulated in the heatmap of DEGs associated with the NOTCH signaling pathway (Figure 8C). JAG1 is expressed in highly proliferative epithelial layers, such as the basal layer of the oral epithelium and the stratum spinosum (58). As one of the NOTCH ligands, JAG1 promotes the activation of NOTCH intracellular domains (NICDs) by facilitating protease activation (59). Numerous studies have confirmed the promotional role of JAG1 and NOTCH signaling pathways in angiogenesis (60, 61). Notably, ERK phosphorylation in tumors is critical for the expression of JAG1 and the activation of the NOTCH signaling pathway (62). Therefore, we selected JAG1 as an additional potential downstream target of CAVIN3 to investigate its pathological role in angiogenesis.

We first demonstrated that the mRNA and protein levels of JAG1 were upregulated under hypoxic conditions, confirming its potential role in a pathological state (Figure 8, D and E). The qPCR and immunoblotting results indicated that the mRNA and protein expression levels of JAG1 were reduced in the CAVIN3-knockdown group (Figure 8, F and G). Additionally, ELISA results from HUVEC supernatants showed decreased JAG1 secretion after CAVIN3 knockdown (Figure 8H). To further demonstrate the regulatory relationship between CAVIN3 and JAG1, we performed JAG1 supplementation on CAVIN3-knockdown HRMECs under hypoxic conditions, which subsequently activated NICDs (Supplemental Figure 8B). Results from scratch (Figure 8I) and tube formation (Figure 8J) experiments indicated that JAG1 supplementation mitigated the inhibitory effect on migration and tube formation of ECs due to CAVIN3 knockdown. Furthermore, in HRMECs transduced with L-CAVIN3, we employed anti-JAG1 treatment to inhibit the action of JAG1 (Supplemental Figure 8C). Upregulation of JAG1 expression in HRMECs transduced with L-CAVIN3 was detected by immunoblotting (Supplemental Figure 9A). Consistent with previous results, we found that the enhanced migration and tube-forming ability due to the overexpression of CAVIN3 could also be inhibited by anti-JAG1 treatment (Supplemental Figure 9, B and C). To further validate the role of ERK phosphorylation in JAG1 expression, we treated HRMECs with PD98059. The results from qPCR and immunoblotting indicated that inhibiting ERK phosphorylation led to decreased JAG1 mRNA and protein levels (Figure 8, K and L). In conclusion, we demonstrated that CAVIN3 deficiency can regulate the process of vascular normalization by inhibiting ERK phosphorylation and subsequently downregulating JAG1 expression in vitro.

### Cavin3 deficiency modulates vascular normalization in an Erk/Jag1-dependent manner.

**Figure 9:**
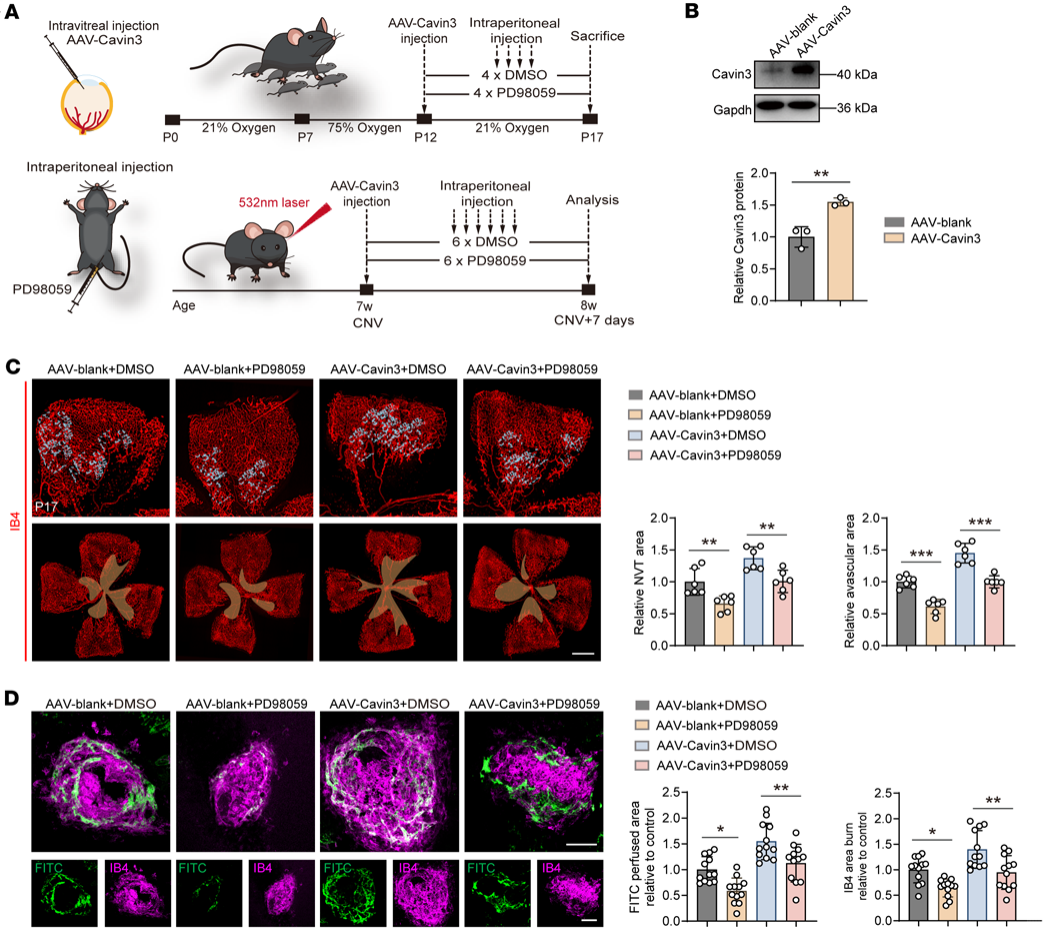
Cavin3 deficiency modulates vascular normalization in an Erk-dependent manner. (**A**) Experimental scheme for **C** and **D**. (**B**) Immunoblotting of Cavin3 in OIR mice injected with AAV-Cavin3 /AAV-blank, using Gapdh as an internal reference. *n* = 3 per group. (**C**) Immunofluorescent staining of IB4 in retinas from DMSO-treated OIR mice injected with AAV-Cavin3/AAV-blank or PD98059-treated OIR mice injected with AAV-Cavin3/AAV-blank on P17. *n* = 6 per group. Blue areas indicate NVT areas, while orange areas mark avascular areas. Scale bar: 50 μm. (**D**) Immunofluorescent staining of IB4 and FITC-dextran in the RPE-choroid-sclera complex of DMSO-treated CNV mice injected with AAV-Cavin3/AAV-blank or PD98059-treated CNV mice injected with AAV-Cavin3/AAV-blank. *n* = 12 burns per group. Scale bars: 100 μm. Data are presented as mean ± SD. *P < 0.05; **P < 0.01; ***P < 0.001 by 2-tailed Student’s *t* test (**B**) or 1-way ANOVA with Tukey’s multiple-comparison test (**C** and **D**).

To further investigate the role of Erk phosphorylation and Jag1 in Cavin3 deficiency–mediated vascular normalization, we designed recombinant adeno-associated viruses (AAVs) containing the *Cavin3* coding sequence and the promoter region of mouse TIE to regulate Cavin3 expression in mouse vascular ECs. Vitreous injection of AAV-Cavin3 was performed on P12 in OIR mice and on the same day as laser induction in CNV mice (Figure 9A). Immunoblotting revealed elevated levels of Cavin3 in the retinas of AAV-Cavin3–transduced OIR mice (Figure 9B). Additionally, immunofluorescent staining of retinal flat mounts demonstrated an upregulation of Cavin3 expression in ECs (stained with IB4) of AAV-Cavin3–transduced OIR mice compared with AAV-blank controls (Supplemental Figure 10). We first administered daily intraperitoneal injections of PD98059 to OIR and CNV mice for 4 and 6 days, respectively (Figure 9A). The upregulation of the area of central retinal NVTs and the avascular zone resulting from Cavin3 overexpression was attenuated by PD98059 treatment in OIR mice (Figure 9C). Consistent findings were also observed in the CNV mouse model. The PD98059 treatment reversed the increase in the CNV area labeled by IB4 and FITC-dextran induced by Cavin3 overexpression (Figure 9D). These results suggest that pathological neovascularization processes exacerbated by Cavin3 overexpression can be alleviated when Erk phosphorylation is inhibited. Thus, we provide strong evidence that Erk phosphorylation is downstream of Cavin3’s regulation of ocular neovascularization.

**Figure 10:**
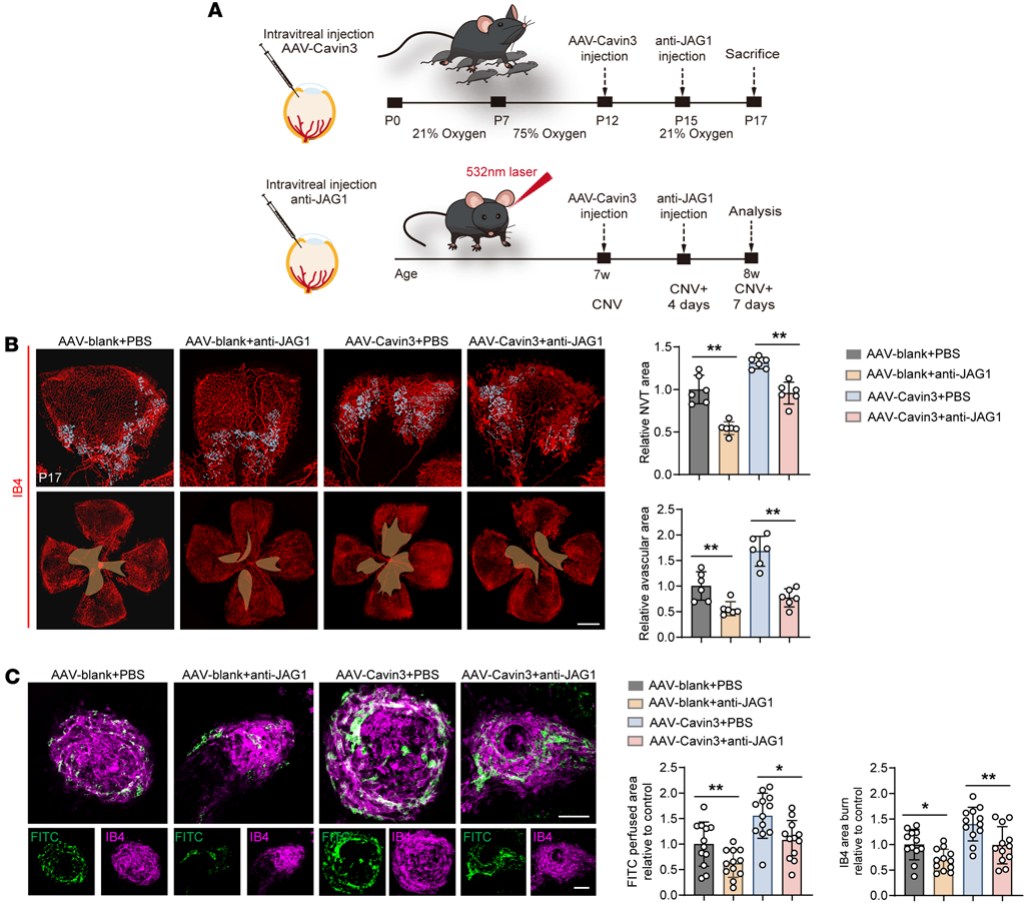
Jag1 is regulated by endothelial Cavin3. (**A**) Experimental protocols for **B** and **C**. (**B**) Immunofluorescent staining of IB4 in retinas from PBS-treated OIR mice injected with AAV-Cavin3/AAV-blank or anti-JAG1–treated OIR mice injected with AAV-Cavin3/AAV-blank on P17. *n* = 6 per group. Blue areas indicate NVT areas, while orange areas mark avascular areas. Scale bars: 50 μm. (**C**) Immunofluorescent staining of IB4 and FITC-dextran in the RPE-choroid-sclera complex of PBS-treated CNV mice injected with AAV-Cavin3/AAV-blank or anti-JAG1–treated CNV mice injected with AAV-Cavin3/AAV-blank. *n* = 12 burns per group. Scale bars: 100 μm. Data are presented as mean ± SD. *P < 0.05; **P < 0.01 by 1-way ANOVA with Tukey’s multiple-comparison test.

We then validated the role of Cavin3 in the regulation of Jag1 in mice. We performed vitreous injections of anti-JAG1 into OIR and CNV mice treated with AAV-Cavin3 on P15 and on day 4 of CNV, respectively (Figure 10A). Our findings indicated that the area of NVTs and the avascular zone expanded by Cavin3 overexpression was reduced following anti-JAG1 treatment in the retinas of OIR mice (Figure 10B). Furthermore, the increased CNV area labeled by IB4 and FITC-dextran in CNV mice injected with AAV-Cavin3 was suppressed following anti-JAG1 treatment (Figure 10C). In conclusion, Erk and Jag1 are critical downstream mediators in the development of pathological angiogenesis in the eye, which is regulated by endothelial Cavin3.

### ZEB1 modulates the expression of CAVIN3.

**Figure 11:**
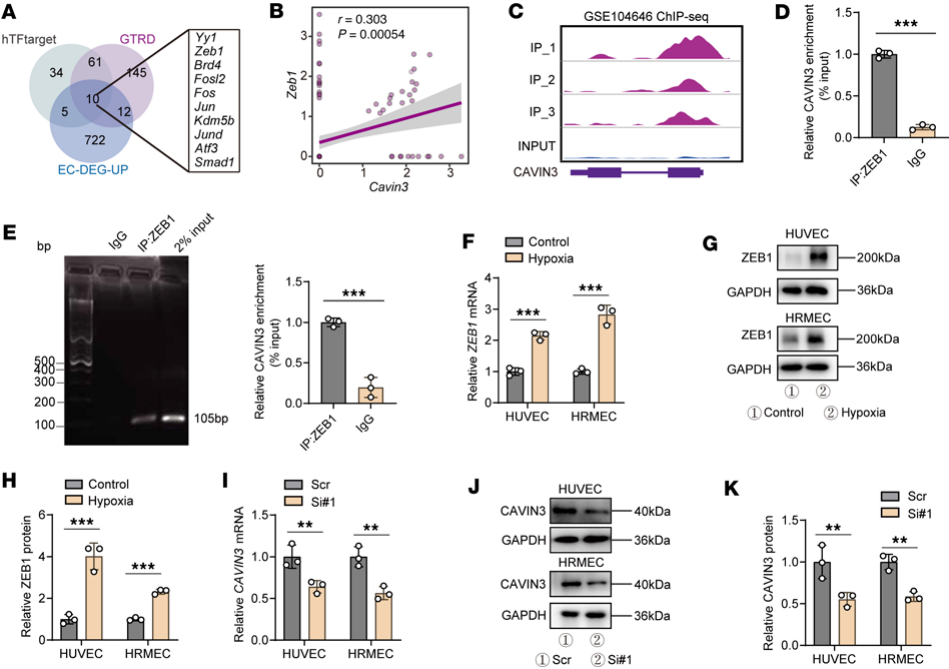
ZEB1 modulates the expression of CAVIN3. (**A**) Combined analysis of hTFtarget, GTRD, and OIR scRNA-seq reveals 10 shared upstream factors that may regulate *Cavin3*. (**B**) Correlation analyses demonstrate the correlation between *Zeb1* and *Cavin3*. (**C**) Ridge plots display the expression in ChIP-seq data (GSE104646) of ZEB1, corresponding to the mRNA coding sequence of *CAVIN3*. (**D**) ChIP-qPCR results indicate that ZEB1 binds to the 5′UTR region of *CAVIN3*. *n* = 3 per group. (**E**) Agarose gel electrophoresis demonstrates the binding of the 5′UTR DNA fragment of ZEB1 to *CAVIN3*, normalized by input. *n* = 3 per group. (**F**) mRNA levels of *ZEB1* in HUVECs and HRMECs after control or hypoxic treatment. *n* = 3 per group. (**G** and **H**) Immunoblotting of ZEB1 in HUVECs and HRMECs following control or hypoxic treatment, using GAPDH as an internal reference. *n* = 3 per group. (**I**) mRNA levels of *CAVIN3* in HUVECs and HRMECs after control treatment (Scr) or ZEB1 knockdown (Si1). *n* = 3 per group. (**J** and **K**) Immunoblotting of CAVIN3 in HUVECs and HRMECs after control treatment or ZEB1 knockdown, using GAPDH as an internal reference. *n* = 3 per group. Data are presented as mean ± SD. **P < 0.01, ***P < 0.001 by 2-tailed Student’s *t* test (**D**–**K**).

We went on to validate the potential upstream regulation of CAVIN3 expression. We co-analyzed 2 open-source transcription factor target gene databases, gene transcription regulation database (GTRD) and hTFtarget, with upregulated DEGs from the OIR scRNA-seq data (GEO GSE150703) (28), and screened 10 shared genes (Figure 11A). Notably, *Zeb1* was upregulated in OIR retinal ECs and exhibited the strongest positive correlation with *Cavin3* (Figure 11B). ZEB1 in the cornea was shown to promote EC proliferation and corneal neovascularization, and its regulatory role was independent of VEGF (63). We therefore next sought to identify ZEB1 as a potential factor upstream of CAVIN3.

ChIP-seq using an anti-ZEB1 antibody (GEO GSE104646) (64) showed that ZEB1 was remarkably enriched in the 5′UTR region of the *CAVIN3* gene (Figure 11C). ChIP-qPCR and agarose gel electrophoresis of the ChIP-PCR product further validated that ZEB1 was enriched in the 5′UTR region of *CAVIN3* (Figure 11, D and E). We next confirmed that ZEB1 promotes CAVIN3 expression under hypoxic conditions. qPCR (Figure 11F) and immunoblotting (Figure 11, G and H) showed that ZEB1 levels were upregulated in hypoxia-treated HRMECs and HUVECs. We selected the ZEB1-siRNA (Si1) with the highest knockdown efficiency to treat HRMECs and HUVECs (Supplemental Figure 5, E–H), and found that CAVIN3 mRNA and protein levels were decreased compared with controls (Scr) (Figure 11, I–K). Taken together, these results show that ZEB1 could positively regulate CAVIN3 expression on the transcriptional level.

## Discussion

Increasing evidence suggests that caveolins and Cavins in ECs play important roles in blood perfusion and blood-brain barrier properties, further regulating angiogenesis and development (19, 65, 66). However, the role and regulatory network of CAVIN3 in angiogenesis has not been fully elucidated. The present study confirmed that CAVIN3 was upregulated in nAMD and PDR. Elevated Cavin3 expression was also detected in the classical retinal/CNV mouse models. We demonstrated the inhibitory effect of Cavin3 deficiency on OIR and CNV, establishing that Cavin3 deficiency further disrupts EC proliferation and vascular sprouting, thereby promoting vascular normalization by partially restoring microenvironmental hypoxia and reestablishing pericyte-EC interactions. Mechanistically, we showed that CAVIN3 deficiency inhibited ERK phosphorylation, which subsequently downregulated JAG1 expression and inhibited NOTCH signaling pathway activation, thereby regulating vascular normalization. At the upstream level, we identified ZEB1 as an upstream regulator of CAVIN3. Our study reveals that targeting CAVIN3 may represent a potential avenue for therapeutic interventions in the treatment of neovascular ocular diseases.

Caveolins and Cavins are the major proteins involved in the formation of caveolae. Caveolins include caveolin 1 (CAV1), CAV2, and CAV3, while Cavins act as scaffolding proteins that remodel membranes into caveolae (65). This family includes CAVIN1, CAVIN2, CAVIN3, and CAVIN4 (65). Recent studies have found that the caveolin and Cavin families can play roles in angiogenesis and remodeling across multiple systems in the body by regulating vascular barrier function, neuroinflammation, and oxidative stress mechanisms. Boopathy et al. reported that CAVIN2 in ECs promotes angiogenesis in vitro and is expressed at elevated levels in the retinas of OIR mice (18). Notably, Hansen et al. detected upregulation of Cavin3 expression in multiple tissues of Cavin2-knockout mice and speculated that Cavin3 may compensate for the angiogenesis-promoting function of Cavin2 (67). This notion was validated in our study, which demonstrated that Cavin3 is involved in pathological ocular neovascularization. Additionally, Liu et al. found that Cav2 can promote tumor angiogenesis (68). However, Cav1 has been reported to play a protective role in antineovascularization and maintenance of vascular permeability in ocular neovascularization, as noted by Jiang et al. (69). Thus, it is essential to determine the regulatory role of CAVIN3 in neovascularization, which could further clarify the distinct roles played by the caveolin and Cavin families in pathological angiogenesis, thereby addressing a substantial research gap. On another note, as the use of vitreous injections of anti-VEGF therapeutic drugs in treating fundus neovascular diseases has gradually increased, challenges such as drug resistance, side effects, and diminished drug efficacy have emerged in patients (9). Therefore, searching for novel targets for antineovascular therapy is urgent. In this study, we conclude that EC CAVIN3 deficiency can inhibit pathological angiogenesis, restore the barrier function of the BRB, and improve microenvironmental hypoxia in the pathological progression of ocular neovascular disease, suggesting a potential therapeutic role. However, due to the complex etiology and intricate course of ocular neovascular diseases, the role of CAVIN3 in the immune microenvironment and oxidative stress needs further investigation in future studies.

To better understand the mechanism of CAVIN3’s role in pathological neovascularization, the present study identified the ERK/JAG1 signaling pathway downstream of CAVIN3. Despite the limited number of previous studies on CAVIN3, its promotion of ERK phosphorylation has been reported multiple times. Hernandez et al. identified that CAVIN3 can enhance ERK phosphorylation by anchoring vesicles containing ERK-activating modules to the cytoplasmic membrane (57). ERK signaling regulates cell proliferation, differentiation, and angiogenesis in both the eye and tumors, and has consistently been implicated as a downstream component of proangiogenic pathways (70). This aligns with our conclusion that ERK is a downstream target of CAVIN3. As a ligand in the NOTCH signaling pathway, Jag1 is a potent proangiogenic regulator in mice that destabilizes stalk cells, leading to the formation of immature vascular plexuses (60). Sharma et al. demonstrated both in vitro and in vivo that enhanced Jag1-mediated activation of Notch1 promotes pathological angiogenesis in proliferative retinopathy (59). In diabetic microangiopathy, endothelial Jag1 has also been shown by Yoon et al. to promote diabetic retinopathy by downregulating VE-cadherin expression (61). Furthermore, Li et al. suggested that ERK phosphorylation positively regulates Jag1 and Notch1 in tumors, which is consistent with our finding that inhibition of ERK phosphorylation reduces JAG1 expression (71). Additionally, there may be other potential downstream targets and pathways for CAVIN3 in ECs. Further in-depth studies on the regulatory mechanisms and pathogenic roles of CAVIN3 in ocular neovascular diseases are warranted.

In terms of mechanistic studies, we also examined the upstream transcriptional regulation of CAVIN3. Jin et al. previously published their findings on the promotional role of ZEB1 in ocular vascular EC proliferation and corneal pathological neovascularization, suggesting that this role is independent of VEGF expression (63, 72). Building on this, we verified that ZEB1 promotes the upregulation of CAVIN3 expression during the pathology of ocular neovascularization. However, since the fusion of tip cells during vascular sprouting exerts shear stress on the vessel wall and CAVIN3 is activated under stress conditions, it remains to be systematically investigated whether other factors could also lead to the upregulation of CAVIN3 expression in pathological contexts.

In summary, our study demonstrated the regulatory role of endothelial CAVIN3 in ocular neovascular disease. Utilizing OIR and CNV models, we further established that CAVIN3 deficiency exerts protective effects in pathological conditions by inhibiting pathological neovascularization, restoring microenvironmental hypoxia, reestablishing the barrier function of the BRB, and preventing the proliferation of pathological vasculature. Additionally, we identified both upstream and downstream targets of endothelial CAVIN3, refining the mechanisms by which CAVIN3 regulates pathological neovascularization. Collectively, this work represents a comprehensive analysis of CAVIN3’s regulatory role in neovascularization, addresses a gap in the study of the Cavin family in this context, and provides insights into the normalization of ocular vasculature, highlighting promising potential targets for the treatment of neovascular ophthalmopathy.

## Methods

Further information can be found in the Supplemental Methods.

### Sex as a biological variable.

Both female and male animals were used in the study, but sex was not considered as a biological variable.

### Cell culture and treatment.

HUVECs (LGC Standards, ATCC-PCS-100-013) were cultured in low-glucose DMEM with 10% FBS (Invitrogen), 100 U/mL penicillin (Invitrogen), and 100 U/mL streptomycin (Invitrogen). HRMECs (Cell Systems Corporation, ACBRI 181) were cultured in high-glucose DMEM with 10% FBS, 100 U/mL penicillin, and 100 U/mL streptomycin. Cells were cultured at 37°C, 21% O_2_, and 5% CO_2_. In the hypoxic treatment of cells, they were collected after incubation at 1% oxygen concentration for 24 hours. For PD98059 treatment, HRMECs were maintained in a complete medium supplemented with PD98059 (10 μM; MedChemExpress) for 24 hours before collection. For JAG1 treatment, HRMECs were maintained in a complete medium supplemented with JAG1 (40 μM; MedChemExpress) for 24 hours before collection. For anti-JAG1 treatment, HRMECs were maintained in a complete medium supplemented with anti-JAG1 antibody (60 μg/mL; R&D Systems, catalog AF1277) for 24 hours before collection. We assessed the drug efficiency of JAG1 and anti-JAG1 treatment by detecting NICD expression 20 minutes after drug administration.

### Cell transduction and transfection.

Empty lentivirus (L-EV) and lentivirus containing the wild-type human *CAVIN3* gene coding sequence (L-CAVIN3) were constructed by Genepharma. Lentiviruses were added to HRMECs with Polybrene (5 μg/mL; Genepharma) at a multiplicity of infection (MOI) of 10. The transduced HRMECs were subsequently selected using puromycin (5 μg/mL; Sigma-Aldrich) for further experiments. HRMECs and HUVECs were transfected using Lipofectamine 3000 transfection reagent (Invitrogen) in transfection experiments. Scramble siRNA and siRNA targeting *CAVIN3* or *ZEB1* were purchased from Genepharma, with sequence details provided in Supplemental Table 1.

### Mouse breeding and manipulations.

C57BL/6J mice purchased from Charles River Laboratories were housed in a pathogen-free facility at Nanjing Medical University and maintained under a 12-hour light/dark cycle at 28.5°C. Embryos were produced through natural mating, and no randomization was performed in this experiment. Before all invasive procedures and examinations, mice were anesthetized with ketamine (80 mg/kg) and xylazine (4 mg/kg) via intraperitoneal injection. Pupils were dilated using 1% cyclopentolate hydrochloride and 2.5% phenylephrine.

Laser photocoagulation of the mouse fundus was conducted using an argon laser (Lumenis, Inc.) with a central wavelength of 532 nm, an incident power of 200 mW, a spot size of 100 μm, and a pulse duration of 100 ms to induce rupture of Bruch’s membrane and prepare a CNV mouse model. The OIR mouse model was established by placing P7 neonatal mice and female mice in a 75% O_2_ airtight chamber for 5 days. Analysis was performed on P17 for the OIR model and on day 7 after CNV modeling.

Intravitreal injections were performed using a 5 μL Hamilton syringe (Hamilton) with a 33-gauge needle to deliver reagent through a scleral incision (1 mm posterior to the superior limbus) into the vitreous. Cavin3-siRNA or scramble siRNA (injection volume, 1 μL; Genepharma) was injected at the time of OIR exit from the chamber (P12) and on the day of CNV modeling, respectively. AAV-blank or AAV-Cavin3 (injection volume, 1 μL; 1 × 10^13^ genome copies/mL; AAV serotype, Vec; EC-specific promoter, TIE; Genepharma) was injected on P12 and on the day of CNV modeling, respectively. Anti-JAG1 (R&D Systems) and PBS were injected on P15 and on day 4 of CNV modeling, respectively.

PD98059 (MedChemExpress) was administered daily via intraperitoneal injection to mice at a dose of 10 mg/kg. The OIR mice received intraperitoneal injections on P13, P14, P15, and P16, while the CNV mice received intraperitoneal injections from day 1 to day 6 of CNV modeling.

### Statistics.

Statistical analyses were performed using Prism (version 10.1.2, GraphPad Software). Comparisons between 2 experimental groups were made using 2-tailed Student’s *t* tests. Statistical significance for differences among groups was tested by 1-way ANOVA with Tukey’s multiple-comparison test. Data represent mean ± SD. A *P* value of less than 0.05 was considered significant. Detailed replication information for each experiment is provided in the figure legends.

### Study approval.

FVMs from PDR patients were obtained from the Department of Ophthalmology at The First Affiliated Hospital of Nanjing Medical University. All procedures followed the Association for Research in Vision and Ophthalmology (ARVO) statement on Human Subjects and the Declaration of Helsinki with written informed consent signed by all individuals before donation. The study was approved and reviewed by the ethics committee of The First Affiliated Hospital of Nanjing Medical University (approval no. 2023-SR-199). Animal experiments that conformed to the NIH *Guide for the Care and Use of Laboratory Animals* (National Academies Press, 2011) were approved and consistently reviewed by the Ethical Review Committee of Nanjing Medical University (approval no. IACUC-2408075).

### Data availability.

RNA-seq data have been deposited in the NCBI GEO database under accession number GSE275653. Values for all data points in graphs are reported in the Supporting Data Values file.

## Author contributions

WL and YZ designed research studies, conducted experiments and acquired and analyzed data. WL, YZ, HZ, NS, and RS performed the investigation and visualization. SY, HZ, and XM supervised the study. WL, HZ, and QY wrote the original draft of the manuscript, which was reviewed and edited by SY, WL, YZ, XM, and QY. All authors read and approved the final manuscript. The order of co–first authors was decided by discussions among the 2 first authors and the corresponding authors.

## Supplementary Material

Supplemental data

Unedited blot and gel images

Supporting data values

## Version 1 05/08/2025

Electronic publication
