# Supplementary material for: CAVIN3 deficiency promotes vascular normalization in ocular neovascular disease via ERK/JAG1 signaling pathway
Source: JCI Insight. 2025 May 8;10(9):e187836. doi: 10.1172/jci.insight.187836 (PMC12128960; doi:10.1172/jci.insight.187836)
Supplement: Supplemental data [file jciinsight-10-187836-s173.pdf]

Supplemental Material

CAVIN3 deficiency promotes vascular normalization in ocular neovascular disease via ERK/JAG1 signaling pathway.

Supplemental materials include 10 Supplemental Figures, 3 Supplemental Tables, and Supplemental Methods.

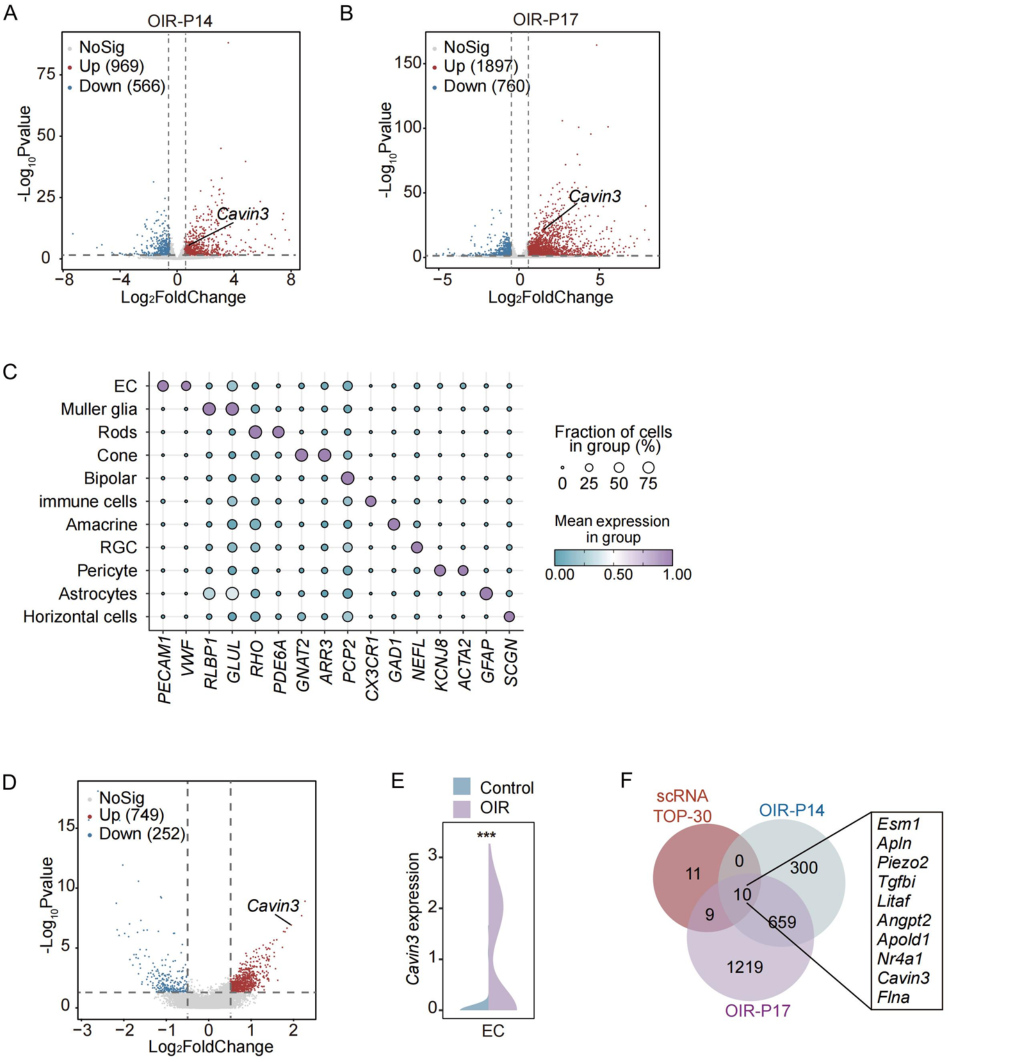

Supplemental Figure 1. Analysis of *Cavin3* expression in RNA-seq data of OIR. (**A** and **B**) OIR-modeled P14 (**A**) and P17 (**B**) mouse retina bulk RNA-seq data (GSE234447) are analyzed for DEGs. (**C**) Each cell type in OIR retinas is identified and classified using classical markers (GSE150703). (**D**) Volcano plots display DEGs in the ECs of OIR mouse retinas (GSE150703). (**E**) A violin plot illustrates *Cavin3* expression in OIR retinal ECs (GSE150703). (**F**) Differential analyses of OIR retinal scRNA-seq and OIR retinal bulk RNA-seq were compared, identifying ten shared differential genes. ***$P < 0.001$.

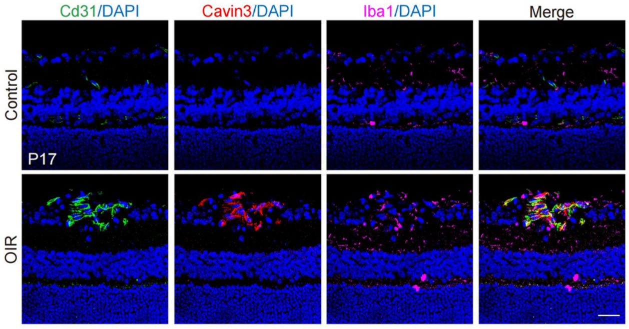

Supplemental Figure 2. Co-localization analysis of Cavin3 in the retina of OIR mice. Immunofluorescence staining (Cavin3, Cd31, and Iba1) in retinal cryosections of control and OIR P17 mice. *n* = 6 per group. Scale bar: 50μm.

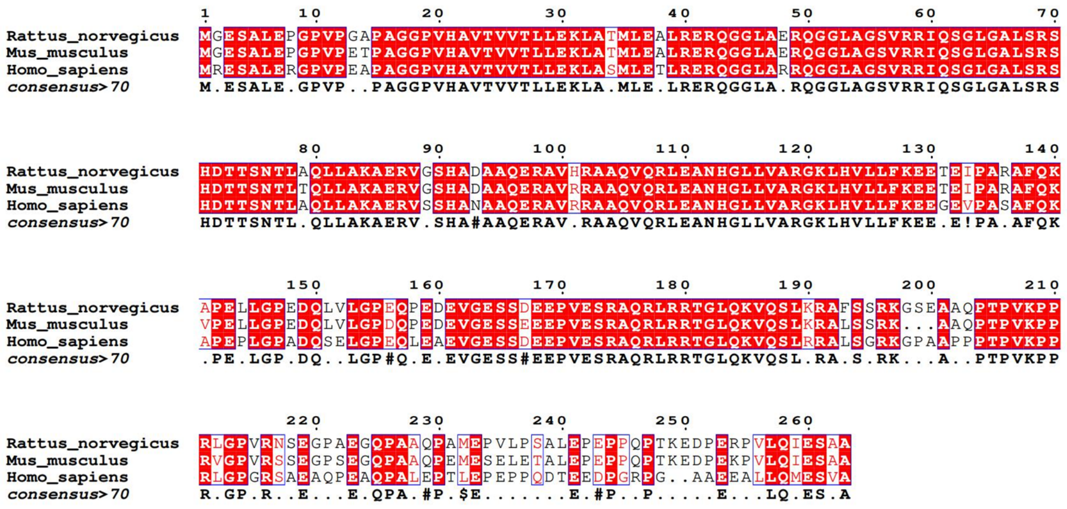

Supplemental Figure 3. CAVIN3 is evolutionarily conserved across different species. The phylogenetic tree is constructed using Evolview, illustrating that various species of CAVIN3 are clustered and classified into distinct subfamilies.

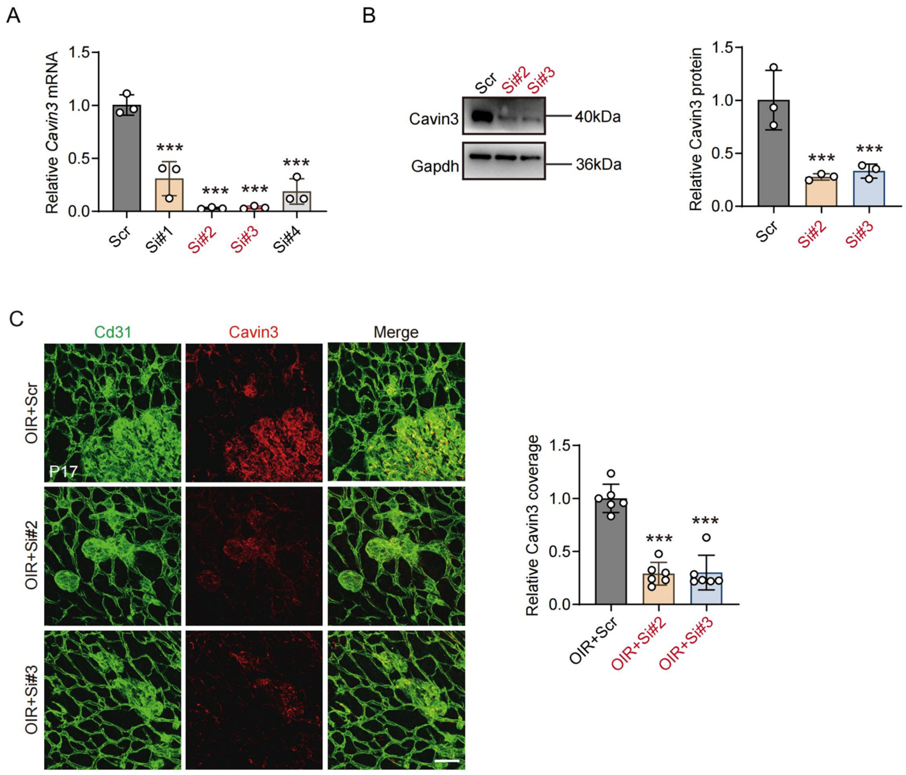

Supplemental Figure 4. The efficiency of siRNA knockdown targeting *Cavin3* in OIR mice. (**A**) mRNA levels of *Cavin3* in different siRNA knockdown retinas from the OIR model. *n* = 3 per group. (**B**) Immunoblotting of Cavin3 in control and Cavin3-siRNA treated OIR retinas, using Gapdh as an internal reference. *n* = 3 per group. (**C**) Immunofluorescence staining of Cd31 and Cavin3 in retinal flat mounts of control and Cavin3-siRNA treated OIR mice. *n* = 6 per group. Scale bar: 50μm. Error bars represent mean ± SD. ***P < 0.001. 2-tailed Student's t tests.

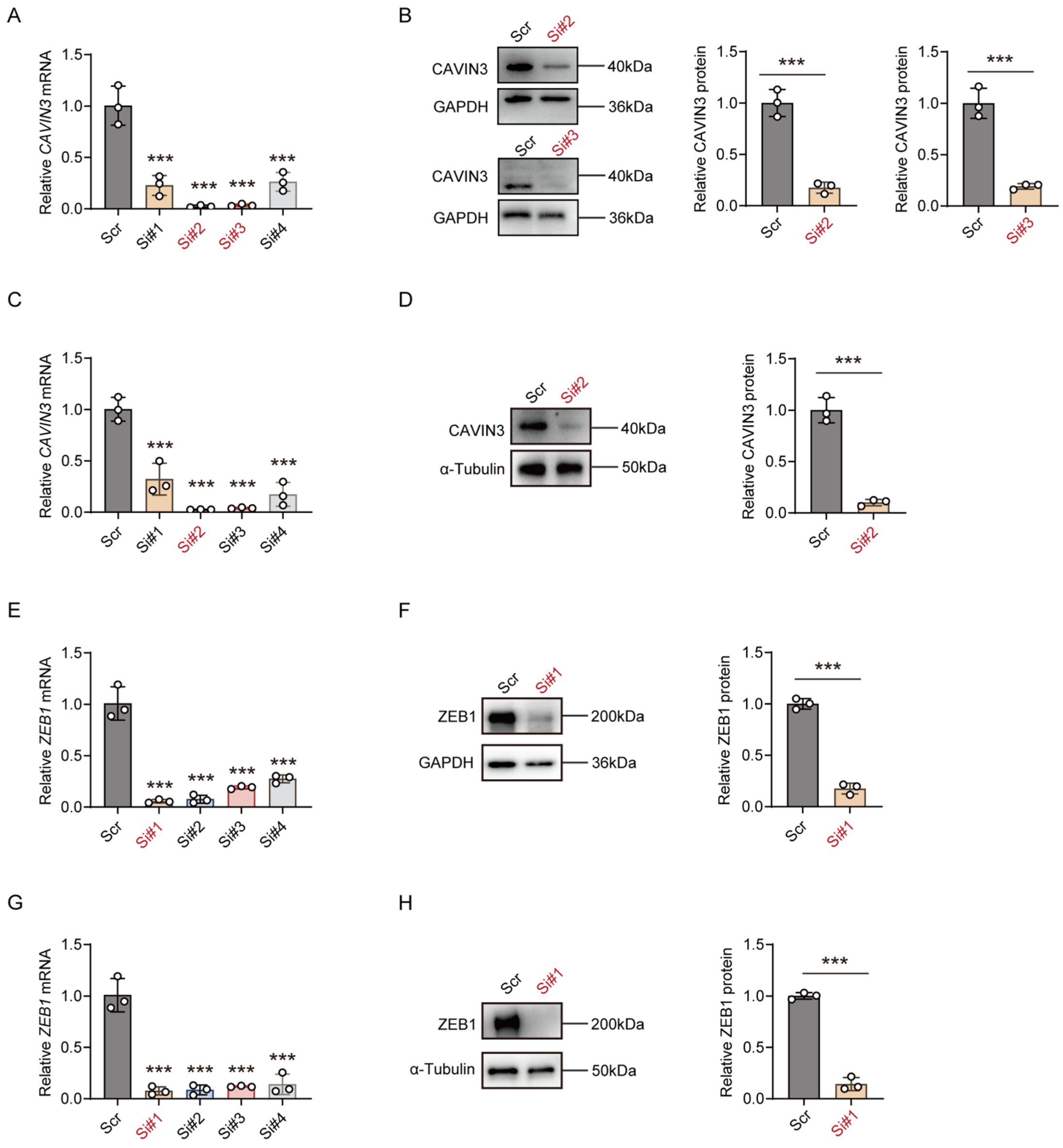

Supplemental Figure 5. The efficiency of siRNA knockdown targeting *CAVIN3* and *ZEB1* in HRMECs and HUVECs. (**A** and **B**) mRNA (**A**) and protein (**B**) levels of CAVIN3 in HRMECs subjected to different siRNA knockdowns. GAPDH is used as an internal reference. n = 3 per group. (**C** and **D**) mRNA (**C**) and protein (**D**) levels of CAVIN3 in HUVECs subjected to different siRNA knockdowns. α-Tubulin is used as an internal reference. n = 3 per group. (**E** and **F**) mRNA (**E**) and protein (**F**) levels of ZEB1 in HRMECs subjected to different siRNA knockdowns. GAPDH is used as an internal reference. n = 3 per group. (**G** and **H**) mRNA (**G**) and protein (**H**) levels of

ZEB1 in HUVECs subjected to different siRNA knockdowns. α-Tubulin is used as an internal reference. $n = 3$ per group. Error bars represent mean ± SD. ***$P < 0.001$. 2-tailed Student's *t* tests.

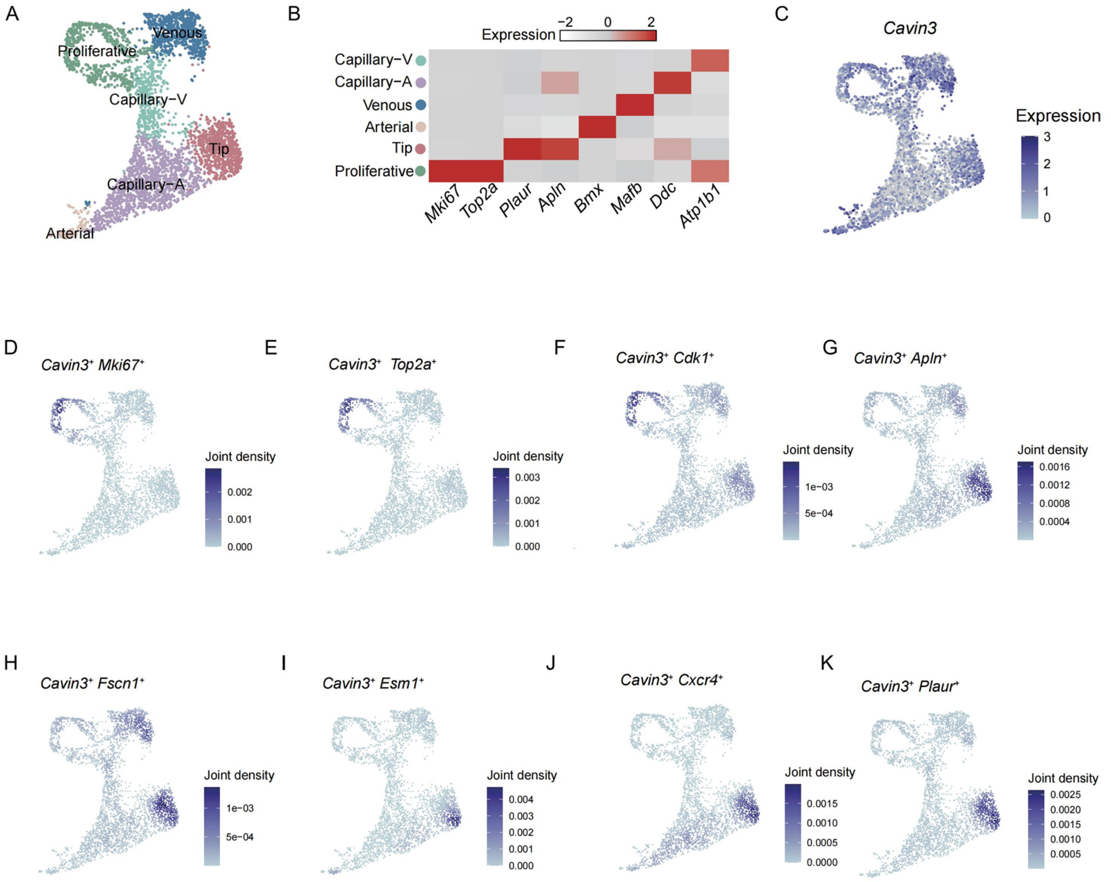

Supplemental Figure 6. Cavin3 has a potential role in promoting the transformation of normal ECs into proliferative ECs and tip cells in developing CNS vascular. (**A**) A UMAP plot illustrates the visualization and subpopulation subgrouping of scRNA-seq (GSE111839) from developing brain endothelial cells. (**B**) A heatmap shows the expression of classical markers across various clusters of developing brain vascular ECs. (**C**) The feature plot displays the distribution of *Cavin3* expression in developing brain vascular ECs. (**D-K**) Feature plots illustrate the co-expression of *Cavin3* with markers in the proliferating and tip ECs.

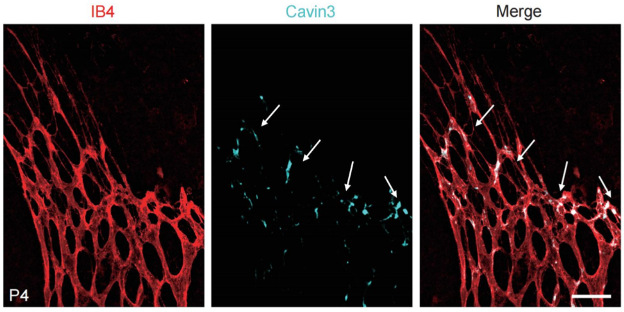

Supplemental Figure 7. Enrichment of Cavin3 in developing mouse retinal vascular tip cells. Immunofluorescence staining of IB4 and Cavin3 in the angiogenic front of the retina in P4 mice. *n* = 6 per group. Scale bar: 50μm.

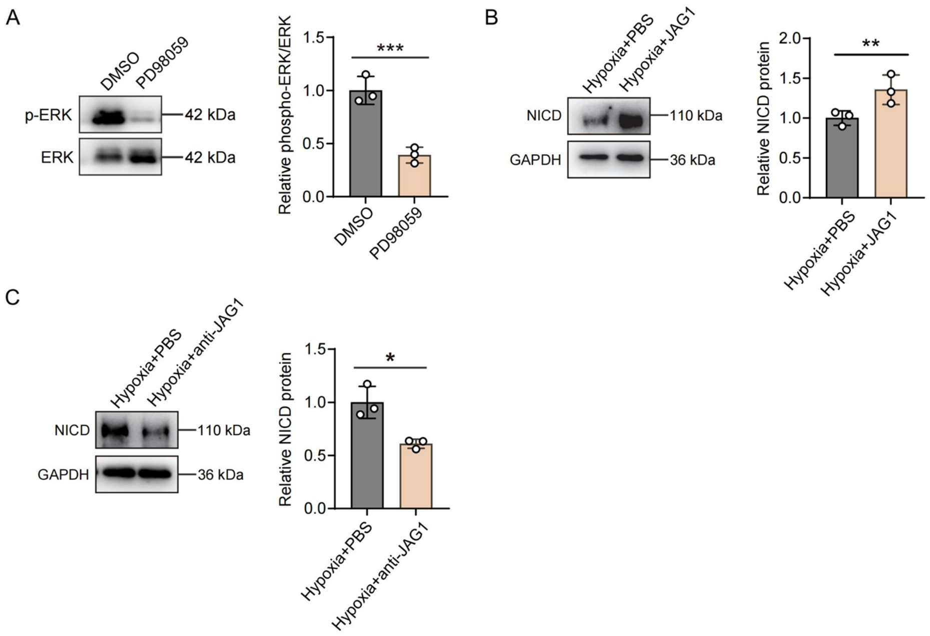

Supplemental Figure 8. The efficiency of treatments in HRMECs. (**A**) Immunoblotting of ERK and phospho-ERK in HRMECs following DMSO or PD98059 treatment, using GAPDH as an internal reference. *n* = 3 per group. (**B**) Immunoblotting of NICD in HRMECs under hypoxia following PBS or JAG1 treatment, using GAPDH as an internal reference. Samples were collected after 20 minutes of treatment. *n* = 3 per group. (**C**) Immunoblotting of NICD in HRMECs under hypoxia following PBS or anti-JAG1 treatment, using GAPDH as an internal reference. Samples were collected after 20 minutes of treatment. *n* = 3 per group. Error bars represent mean ± SD. *$P < 0.05$; **$P < 0.01$; and ***$P < 0.001$. 2-tailed Student's *t* tests.

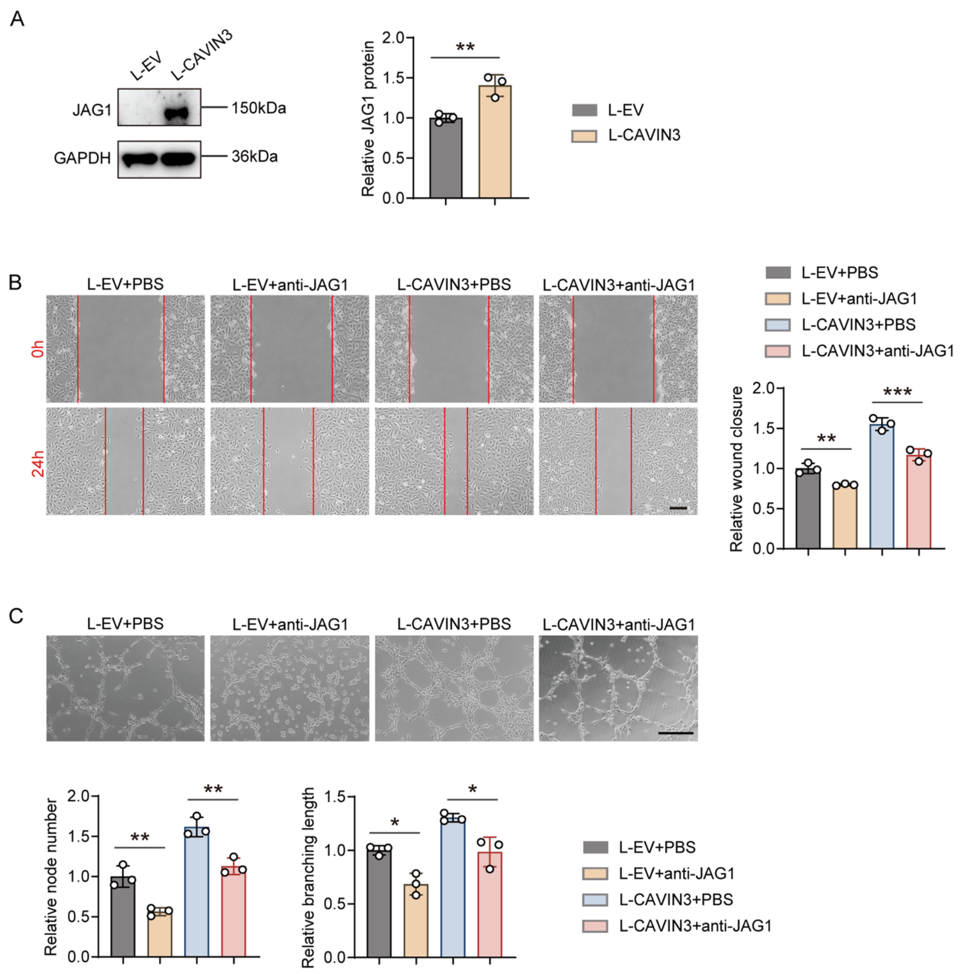

Supplemental Figure 9. Anti-JAG1 suppresses the effects of CAVIN3 on endothelial phenotypes. (**A**) Immunoblotting of JAG1 in HRMECs transduced with L-EV/L-CAVIN3, using GAPDH as an internal reference. n = 3 per group. (**B**) Scratch test on PBS-treated HRMECs transduced with L-EV/L-CAVIN3 or anti-JAG1-treated HRMECs transduced with L-EV/L-CAVIN3. n = 3 per group. Scale bars: 200μm. (**C**) Tube formation assay on PBS-treated HRMECs transduced with L-EV/L-CAVIN3 or anti-JAG1-treated HRMECs transduced with L-EV/L-CAVIN3. n = 3 per group. Scale bars: 100μm. Error bars represent mean ± SD. *P < 0.05; **P < 0.01; and ***P < 0.001. 2-tailed Student's t tests (**A**) and 1-way ANOVA with Tukey's multiple comparisons test (**B** and **C**).

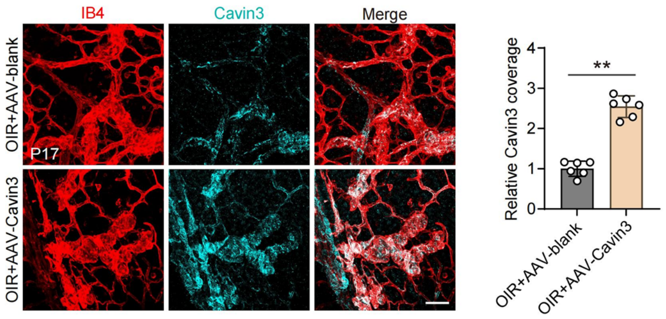

Supplemental Figure 10. The efficiency of AAV overexpression targeting *Cavin3* in OIR mice. Immunofluorescence staining of IB4 and Cavin3 in retinal flat mounts of OIR mice injected with AAV-Cavin3/AAV-blank at P17. $n = 6$ per group. Scale bar: 50μm. Error bars represent mean ± SD. **$P < 0.01$. 2-tailed Student's t tests.

Supplemental Table S1. Sequences of siRNAs.

Targeted genes	Sense sequence (5'→3')	Antisense sequence (5'→3')
In vitro		
scramble siRNA	UUCUCCGAACGUGUCACGUTT	ACGUGACACGUUCGGAGAATT
CAVIN3-siRNA1	ACGCCGUGACGGUGGUGACTT	GUCACCACCGUCACGGCGUTT
**CAVIN3-siRNA2**	GGAAGCUCCACGUUCUGCUTT	AGCAGAACGUGGAGCUUCCTT
**CAVIN3-siRNA3**	GCGCUCUGAGUCGCAGCCATT	UGGCUGCGACUCAGAGCGCTT
CAVIN3-siRNA4	AGGCCUGGCUCGAAGGCAGTT	CUGCCUUCGAGCCAGGCCUTT
**ZEB1-siRNA1**	GGCGGUAGAUGGUAAUGUATT	UACAUUACCAUCUACCGCCTT
ZEB1-siRNA2	GUCGCUACAAACAGUUGUATT	UACAACUGUUUGUAGCGACTT
ZEB1-siRNA3	CUCUGAAAGAACACAUUAATT	UUAAUGUGUUCUUUCAGAGTT
ZEB1-siRNA4	GUGCAGAAGAGCUCUCAAATT	UUUGAGAGCUCUUCUGCACTT
In vivo		
scramble siRNA	UUCUCCGAACGUGUCACGUTT	ACGUGACACGUUCGGAGAATT
Cavin3-siRNA1	CAGAGGAUGAAGUUGGAGATT	UCUCCAACUUCAUCCUCUGTT
**Cavin3-siRNA2**	GGGCUCUUUCCAGUCGUAATT	UUACGACUGGAAAGAGCCCTT
**Cavin3-siRNA3**	GCUUCAAAUAGAGAGCGCATT	UGCGCUCUCUAUUUGAAGCTT
Cavin3-siRNA4	GAAGCUGCACGUCCUGCUCTT	GAGCAGGACGUGCAGCUUCTT

Supplemental Table S2. Primers used in this study.

Gene/RNA	Forward primer (5'→3')	Reverse primer (5'→3')
In vitro		
ACTB	ACCTTCTACAATGAGCTGCG	CCTGGATAGCAACGTACATGG
CAVIN3	CACGTTCTGCTCTTCAAGGAG	TGTACCTTCTGCAATCCGGTG
TOP2A	ACCATTGCAGCCTGTAAATGA	GGGCGGAGCAAAATATGTTCC
MKI67	GACTTTGGGTGCGACTTGAC	ACAACTCTTCCACTGGGACG
CDK1	CCTATGGAGTTGTGTATAAGGGT	AGCACATCCTGAAGACTGACT
ESM1	ACAGCAGTGAGTGCAAAAGCA	GCGGTAGCAAGTTTCTCCCC
PLAUR	TGTAAGACCAACGGGGATTGC	AGCCAGTCCGATAGCTCAGG
CXCR4	ACTACACCGAGGAAATGGGCT	CCACAATGCCAGTTAAGAAGA
APLN	GTCTCCTCCATAGATTGGTCTGC	GGAATCATCCAAACTACAGCCAG
FSCN1	CTGCTACTTTGACATCGAGTGG	GGGCGGTTGATGAGCTTCA
ZEB1	AAGTGGCGGTAGATGGTAATG	AGGAAGACTGATGGCTGAAATAA
JAG1	TGCCAAGTGCCAGGAAGT	GCCCCATCTGGTATCACACT
In vivo		
Gapdh	TGCACCACCAACTGCTTAG	GGATGCAGGGATGATGTTC
Cavin3	CCACCAGCAACACACTGACG	GGAATTTCAGTCTCCTCCTTGAAGA

Supplemental Table S3. Antibodies used in this study.

Anti-protein	Host	Dilution and Application	Supplier	Catalog
In vitro				
GAPDH	Mouse	1:10000, Immunoblotting	Proteintech	60004-1-Ig
CAVIN3	Rabbit	1:2000, Immunoblotting	Proteintech	16250-1-AP
ZEB1	Rabbit	1:2000, Immunoblotting	Proteintech	21544-1-AP
JAG1	Mouse	1:5000, Immunoblotting	Proteintech	66890-1-Ig
β-Actin	Mouse	1:10000, Immunoblotting	Proteintech	66009-1-Ig
α-Tubulin	Rabbit	1:10000, Immunoblotting	Proteintech	11224-1-AP
phosphor-ERK	Rabbit	1:2000, Immunoblotting	Cell Signaling Technology	9101S
ERK	Mouse	1:2000, Immunoblotting	Cell Signaling Technology	4696S
NICD	Sheep	1:200, Immunoblotting	R&D systems	AF3647
In vivo				
Gapdh	Mouse	1:10000, Immunoblotting	Proteintech	60004-1-Ig
		1:100, Immunofluorescence;		
Cavin3	Rabbit	1:2000, Immunoblotting	Proteintech	16250-1-AP
VE-cadherin	Rat	1:75, Immunofluorescence	Abcam	ab282277
Ng2	Rabbit	1:100, Immunofluorescence	Merck	AB5320
Col4	Rabbit	1:150, Immunofluorescence	Abcam	ab6586
Iba1	Rat	1:100, Immunofluorescence	Abcam	AB283346
IB4	/	1:200, Immunofluorescence	Vector Laboratories	FL-1201-.5
Cd31	Hamster	1:200, Immunofluorescence	Merck	MAB1398Z
β-Actin	Mouse	1:10000, Immunoblotting	Proteintech	66009-1-Ig
α-Tubulin	Rabbit	1:10000, Immunoblotting	Proteintech	11224-1-AP

Supplemental Methods

ScRNA-seq and analysis. Single-cell data were analyzed for dimension reduction and clustered using the R package Seurat (version 4.3.0). The OIR and Control datasets were integrated with the R package Harmony (version 1.2.0) to eliminate batch effects. Differential expression analysis was performed using the FindMarkers and FindAllMarkers functions. Pseudotime trajectory was conducted using the R packages Monocle 2 (version 2.28.0) and Slingshot (version 2.12.0). Endothelial cells were sorted in pseudotime using the DDRTree and orderCells functions. Violin plots were visualized using the R package dittoSeq (version 1.12.1). Correlation analysis was performed by calculating Pearson correlations between genes of interest using the R package stats (version 4.3.1) and visualized using the online visualization website Chiplot ('https://www.chiplot.online/#').

Bulk RNA-seq and analysis. High-quality RNA was extracted according to the manufacturer's recommendations. RNA-Seq libraries were subsequently sequenced using the Illumina NovaSeq 6000 platform (Illumina), yielding 150 base pair (bp) sequences. Quality control and raw sequencing data filtering were performed using Fastp (version 0.23.2) and sortmerna (version 4.3.4) software. The STAR software (version 2.7.10) was then used to align the clean sequences with the human genome (GRCh38). Counts and transcripts per million (TPM) were calculated for each gene using Salmon (version 1.9.0).

DESeq2 (version 1.34.0) was employed to identify differentially expressed genes, with differences considered statistically significant at (P < 0.05). Gene Ontology (GO) analysis and pathway enrichment analysis were conducted on the differentially expressed mRNA. The RNA-Seq data have been deposited in the GEO database under accession number GSE275653.

GO analysis. GO analysis of DEGs in RNA-seq was performed using the R package clusterProfiler (version 4.8.3). P < 0.05 for enriched GO pathways was considered statistically significant.

Mounting of mice retina/RPE-choroid-sclera complex. Mice were anesthetized and euthanized, and the eyeballs were removed, with their connective tissue trimmed. For retinal flat mount staining, the anterior segment of the eye and the vitreous body were carefully clipped, and the eyeballs were then placed in 4% paraformaldehyde (PFA) for fixation at room temperature for 1 hour. After washing with PBS, the neural retina was carefully separated from the posterior eye cup and trimmed to form a four-leaf clover retinal flat mount. For RPE-choroid-sclera complex flat mount staining, the entire eye was placed in 4% PFA and fixed at 4°C overnight. After washing with PBS, the anterior segment of the eye and the vitreous body were carefully excised. The RPE-choroid-sclera complex was isolated and trimmed into four-leaf clover-shaped flat mounts.

Frozen eyes sections. Mouse eyeballs were removed and fixed in FAS eyeball fixative (Servicebio, Wuhan, China) at 4°C for 1 h. After washing with

PBS, they were transferred to 10%, 20%, and 30% sucrose solutions for gradient dehydration, where they were left in 30% sucrose at 4°C overnight. The eyes were subsequently embedded with optimal cutting temperature compound (Sakura, Torrance, California, USA) and frozen at -80°C. This was followed by sagittal cryosections of the eyeballs with a thickness of 10μm, and the sections were adhered to slides.

Ophthalmic examinations. Seven days after the induction of CNV, mice were anesthetized, and their pupils were dilated for fundus examination. FFA was performed following an intraperitoneal injection of sodium fluorescein (International Medication Systems, South El Monte, CA, USA) at a dose of 2 μL per gram of body weight. Fluorescent fundus images were acquired using the Heidelberg Retinal Angiography 2 (Heidelberg Engineering, Heidelberg, Germany).

FITC-dextran evaluating choroidal neovascularization. Seven days after the induction of CNV, 0.5 mL of FITC-dextran (Merck, Darmstadt, Germany; average molecular weight: 2000kDa) was perfused into the hearts of anesthetized mice, which were subsequently euthanized. After the eyeballs were removed and fixed in 4% PFA at 4°C overnight, the RPE-choroid-sclera complex was isolated, and flat mounts were prepared. The tissues were incubated in a blocking solution consisting of 3% BSA and 0.3% Triton X-100 for 1 hour, followed by overnight incubation with IB4 (1:100, Vector Laboratories). After washing with PBS, the sections were mounted on slides

and photographed using a confocal microscope (Carl Zeiss Jena, Germany). The fluorescence results were evaluated using ImageJ software.

Hypoxyprobe Staining. The hypoxic status of the retina was assessed using the Hypoxyprobe RedAPC Kit (Hypoxyprobe, Inc., Burlington, Massachusetts, USA) according to the manufacturer ' s instructions. Hypoxyprobe was injected intraperitoneally into P17 OIR mice at a dose of 2.5 mg each, one hour before sampling. The sampled eyes were fixed, and the neural retinas were isolated to prepare flat mounts. The tissues were incubated in a blocking solution consisting of 3% BSA and 0.3% Triton X-100 for one hour, followed by overnight incubation with RED APC dye-MAb1 (1:100, Hypoxyprobe) and IB4 (1:100, Vector Laboratories). After washing with PBS, the sections were mounted on slides. Fluorescence was observed using an inverted microscope (DMi8, Leica, Wetzlar, Germany) equipped with a THUNDER imaging system (Leica). The fluorescence results were evaluated using ImageJ software.

Cell cycle analyses. The cell cycle was analyzed using the Cell Cycle Staining Kit (Multisciences Biotech, Hangzhou, China) according to the manufacturer's instructions. Collected cells were incubated in a DNA staining solution containing an osmotic agent for 30 minutes at room temperature, protected from light. The cell cycle was assessed using a flow cytometer (Beckman Coulter, Brea, CA, USA), and data were analyzed with FlowJo software. A total of 10,000 cells per sample were collected for analysis.

EdU assay. Cell proliferation was assessed using the EdU Apollo567 Kit (RiboBio, Guangzhou, China) according to the manufacturer's instructions. Cells were incubated for 2 hours in EdU solution, fixed in 4% PFA, permeabilized with 0.5% Triton X-100, and fluorescently labeled in Apollo solution. The red signals of proliferating cells were observed using an inverted microscope (DMi8, Leica) equipped with a THUNDER imaging system (Leica).

Transwell migration assay. Cell migration was assessed using transwell chambers with an 8μm pore size. Treated cells were inoculated into the upper chamber containing culture medium and collected after migrating for 24 hours. Cells that migrated to the lower chamber were fixed and stained with crystal violet (Beyotime, Shanghai, China) for 15 minutes. Five different views of each chamber were randomly selected and averaged using an ECLIPSE Ts2 inverted microscope (Nikon, Tokyo, Japan).

Scratch test. HRMECs were scratched in a straight line using the tip of a pipette gun to create a wound. After carefully washing with PBS to remove cellular debris, the culture medium was reintroduced. A line was drawn to mark the outer edge of the culture well plate. Based on these markings, images of the same scratch site were captured immediately after the scratch and 24 hours later using an ECLIPSE Ts2 inverted microscope.

Tube formation assay. HRMECs were inoculated in de-growth factor matrix gel (BD Biosciences, New Jersey, USA) 24-well plates for tube-forming capacity analysis. Images were captured 6 hours after inoculation using an

ECLIPSE Ts2 inverted microscope. Data were analyzed using ImageJ software to count the number of nodes and measure the branch lengths of HRMECs in tube formation.

ELISA. The levels of JAG1 in the cell supernatants were measured using the Human Jagged-1 Protein Enzyme-Linked Immunoassay Kit (CUSABIO, Wuhan, China), following the manufacturer's instructions. Absorbance at 450 nm was recorded using a Multiskan FC plate spectrophotometer (Thermo Fisher Scientific).

RNA isolation and qPCR. Total RNA was extracted from cell lysates and mouse retinas using TRIzol reagent (Invitrogen). RNA concentration and purity were assessed using a Nano-Drop ND-1000 spectrophotometer (Nano-Drop Technologies). The PrimeScript RT kit (Takara, Otsu) was utilized for cDNA amplification. QPCR was performed using ChamQ Universal SYBR qPCR Master Mix (Vazyme, Q711) on a Roche LightCycler 480 II. Detailed information regarding the primers used is provided in Supplemental Table S2.

Immunoblotting. Cells, retinas, and RPE-choroid-sclera complexes were collected and lysed in RIPA lysate (Beyotime) supplemented with PMSF Solution (Beyotime) and phosphatase inhibitors (Beyotime). The prepared proteins were separated by gel electrophoresis and transferred onto polyvinylidene fluoride membranes (Millipore). After sealing, the membranes were incubated overnight at 4°C with the primary antibody (Supplemental Table S3). Subsequently, the membranes were incubated with HRP-coupled

secondary antibody (dilution: 1:10,000) at room temperature for one hour. Blots were captured using a Tanon-5200 Multi chemiluminescent imaging system. Protein quantification was performed using ImageJ software.

Immunofluorescence staining. FVMs, eye-frozen sections, retinal flat mounts, and RPE-choroid-sclera flat mounts were incubated in a blocking solution consisting of 3% BSA and 0.3% Triton X-100 for one hour. Following this, the samples were incubated overnight at 4°C with primary antibodies (Supplemental Table S3) and then with the corresponding fluorescence-coupled secondary antibodies (1:500) for two hours at room temperature. DAPI (Sigma-Aldrich) was used for re-staining the cell nuclei. An inverted microscope (DMi8, Leica) equipped with a THUNDER imaging system (Leica) was used to observe fluorescence. Fluorescence intensity was quantified using ImageJ software.

ChIP-qPCR. ChIP assays were performed using the SimpleChIP® Enzymatic Chromatin IP Kit (Cell Signaling Technology, Massachusetts, USA) according to the manufacturer's instructions. The collected cells were cross-linked with 1% formaldehyde, sonicated, and digested to obtain suitable chromatin fragments. The DNA fragments were collected and incubated overnight at 4°C with ZEB1 antibody. The DNA fragments enriched for binding to ZEB1 were evaluated using qPCR and analyzed after normalization based on the input group.

Agarose gel electrophoresis. The DNA fragments enriched for binding to

ZEB1 after the ChIP assay were subjected to a PCR reaction. The diluted PCR products and DNA ladder (Beyotime) were added to the wells of a 2% agarose gel and electrophoresed at 150 V for 30 minutes at room temperature. Imaging was acquired using the Pro-30 Gel Imaging System (Bio-Rad, California, USA). DNA quantification was performed using Image Lab and ImageJ software.
