# Supplementary material for: CAVIN3 deficiency promotes vascular normalization in ocular neovascular disease via ERK/JAG1 signaling pathway
Source: JCI Insight. 2025 May 8;10(9):e187836. doi: 10.1172/jci.insight.187836 (PMC12128960; doi:10.1172/jci.insight.187836)

Full unedited gel for Figure 2F

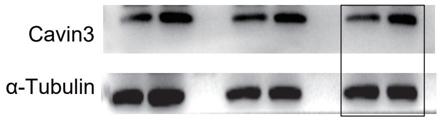

Full unedited gel for Figure 2L

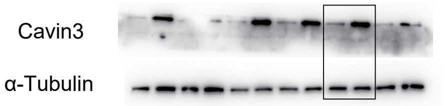

Full unedited gel for Figure 2P

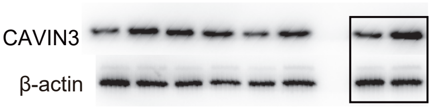

Full unedited gel for Figure 7A

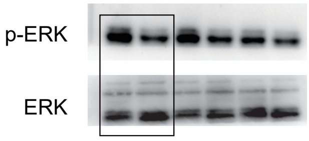

Full unedited gel for Figure 7B

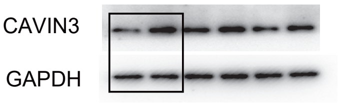

Full unedited gel for Figure 7C

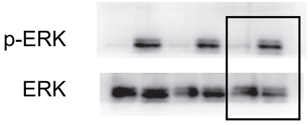

Full unedited gel for Figure 8E

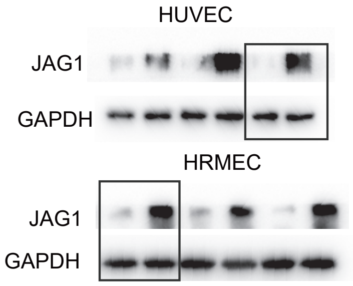

Full unedited gel for Figure 8G

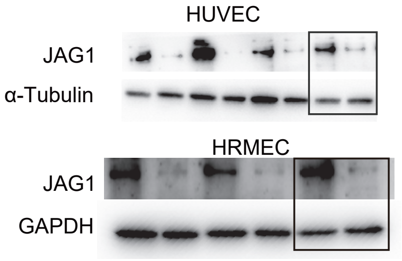

Full unedited gel for Figure 8L

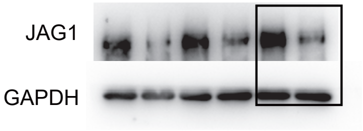

Full unedited gel for Figure 9B

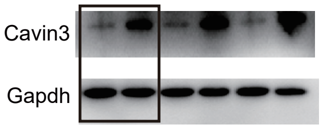

Full unedited gel for Figure 10G

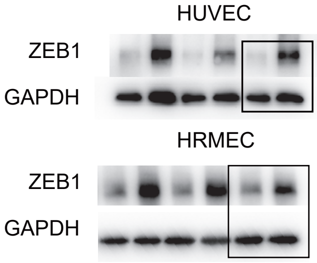

Full unedited gel for Figure 10J

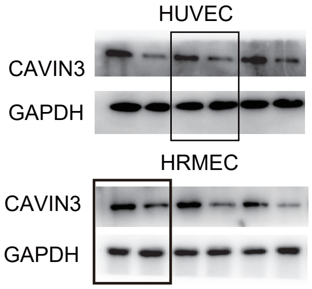

Full unedited gel for Figure S4B

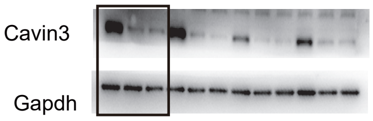

Full unedited gel for Figure S5B

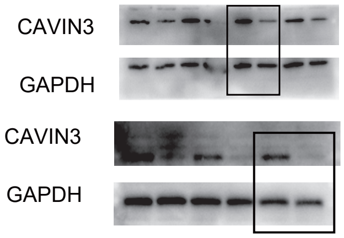

Full unedited gel for Figure S5D

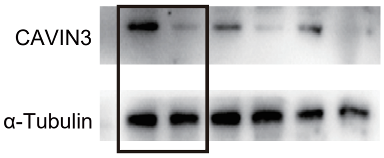

Full unedited gel for Figure S5F

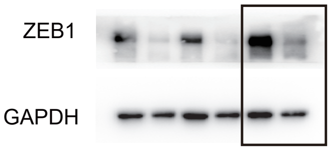

Full unedited gel for Figure S5H

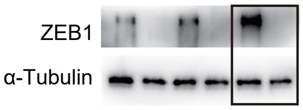

Full unedited gel for Figure S8A

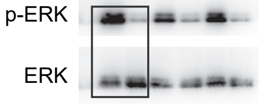

Full unedited gel for Figure S8B

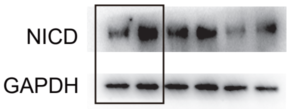

Full unedited gel for Figure S8C

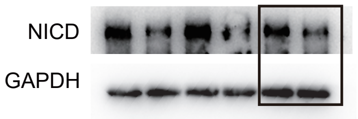

Full unedited gel for Figure S9A

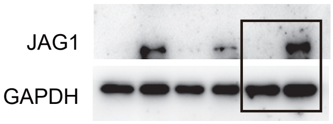

Supplement: Unedited blot and gel images [file jciinsight-10-187836-s174.pdf]
